# Fairness across domains: a unified fairness-aware framework for domain generalization and unsupervised adaptation

**DOI:** 10.3389/fdata.2026.1764468

**Published:** 2026-05-11

**Authors:** Kai Jiang, Chen Zhao, Haoliang Wang, Xintao Wu, Latifur Khan, Christan Grant, Feng Chen

**Affiliations:** 1 Department of Computer Science, The University of Texas at Dallas, Richardson, TX, United States; 2 Department of Computer Science, Baylor University, Waco, TX, United States; 3 Department of Electrical Engineering and Computer Science, University of Arkansas, Fayetteville, AR, United States; 4 Department of Computer & Information Science & Engineering, University of Florida, Gainesville, FL, United States

**Keywords:** domain adaptation, domain generalization, fairness-aware machine learning, machine learning, unsupervised domain adaptation

## Abstract

Fairness in machine learning remains a critical challenge, particularly in the presence of domain shift. We propose a unified fairness-aware framework for both domain generalization (DG) and unsupervised domain adaptation (UDA), which jointly addresses domain shift and sensitive-attribute bias through disentangled representation learning. The framework disentangles content, style, and sensitive factors, and uses them to generate augmented samples that reduce bias while maintaining predictive reliability. Extensive experiments on four datasets demonstrate that the proposed method achieves state-of-the-art performance in both DG and UDA settings. Moreover, it yields a stronger balance between classification accuracy and fairness across diverse domains and sensitive subgroups. By incorporating unlabeled target-domain data, our framework extends prior fairness-aware approaches that were limited to DG and provides new insight into fairness-aware learning under unsupervised adaptation. Overall, this work offers a practical step toward scalable and robust fairness-aware learning in multi-domain environments.

## 1 Introduction

**Figure 1:**
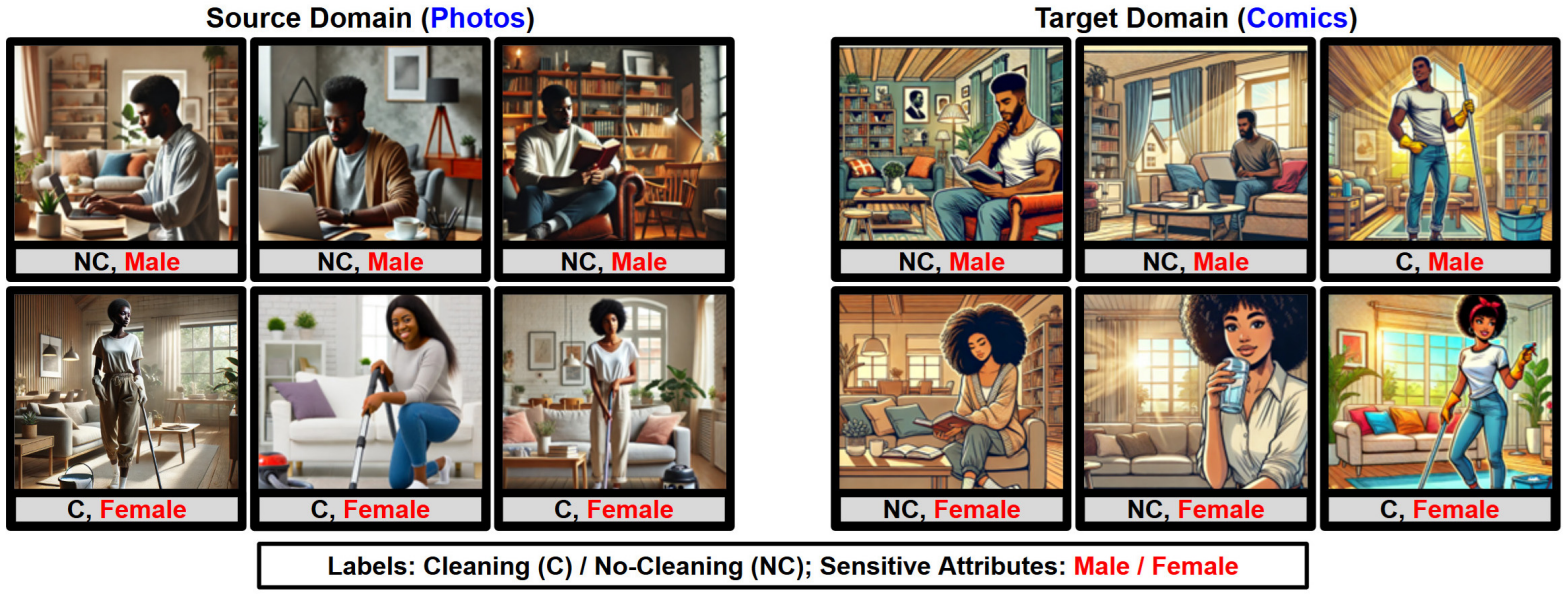
Images in the source and target domains exhibit distinct styles (Photos and Comics). Each domain is characterized by a unique correlation between class labels (NC and C) and sensitive attributes (male and female). Source images taken from FairFace Dataset Karkkainen and Joo (2021).

Ensuring fairness in machine learning has emerged as a cornerstone of ethical AI deployment, especially in real-world applications where domain shifts interact with sensitive attributes to influence prediction outcomes. As machine learning systems increasingly permeate diverse societal domains, fairness considerations become paramount, requiring robust methodologies capable of addressing biases while maintaining prediction performance. Figure 1 illustrates the variations between the source and target domains arising from distinct image styles (Photos and Comics) and correlations between labels (No-cleaning and Cleaning) and sensitive attributes (Male and Female). In the source domain, most women in the kitchen are associated with cleaning, whereas in the target domain, the pattern shifts, and most women are associated with not cleaning. Such changes create a hybrid shift in which both domain-specific style and label-sensitive correlations vary, making it difficult to learn a classifier that is simultaneously accurate and fair.

**Figure 2:**
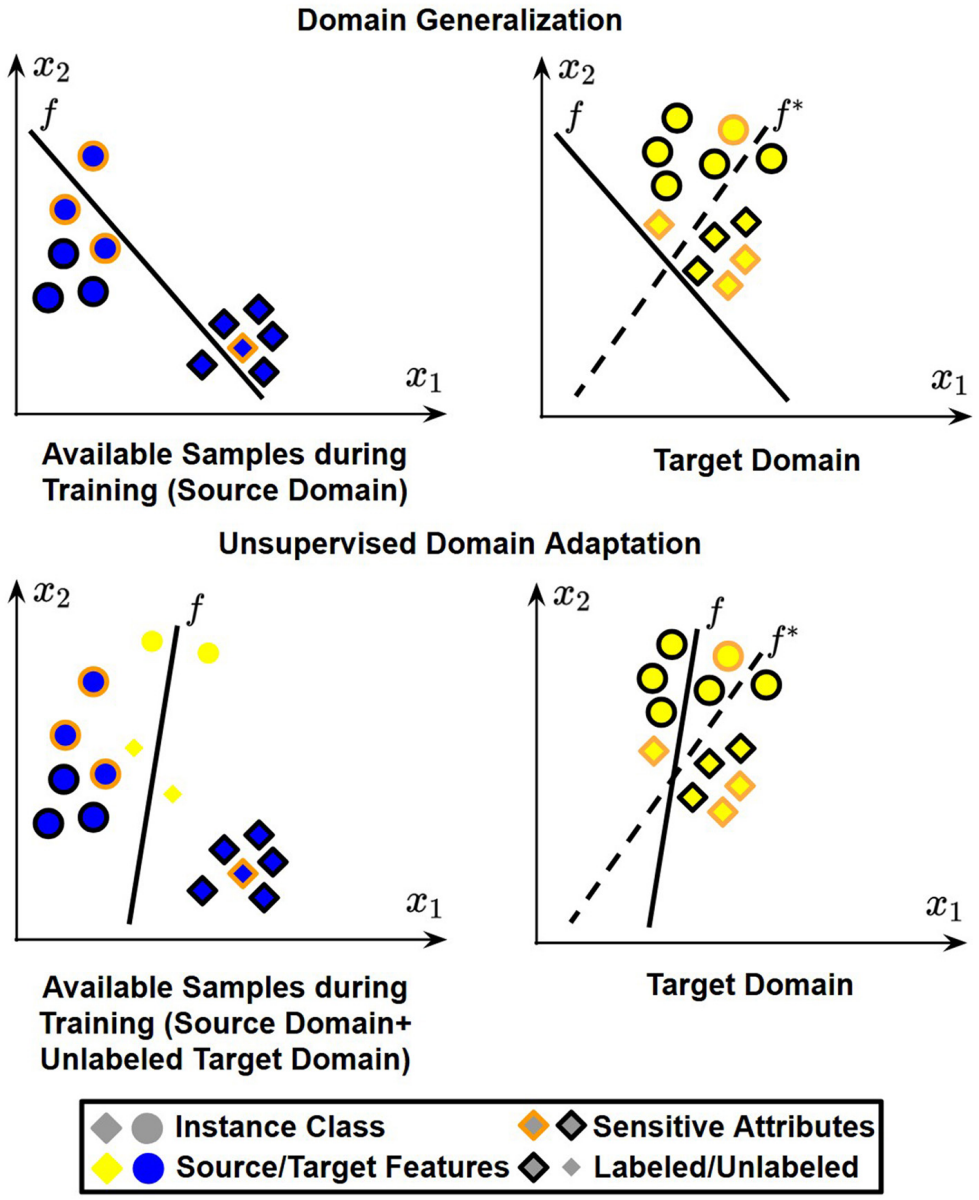
We consider a simple example where $\text{x}={\left[x_{1},x_{2}\right]}^{T}$ is a two-dimensional feature vector. In domain generalization, only labeled samples from source domains are available. In unsupervised domain adaptation, labeled samples from source domains and some unlabeled samples from the target domain are available. A fair classifier *f*, trained on available samples, is applied to data sampled from different types of shifted target domains, leading to misclassification and fairness violations. The optimal classifier is denoted as f^*^.

Two major paradigms for learning under distribution shift are Domain Generalization (DG) and Unsupervised Domain Adaptation (UDA). DG aims to learn from *labeled data from multiple source domains* and generalize to an unseen target domain without access to target-domain data during training, as illustrated in the top part of Figure 2. UDA, in contrast, assumes access to *unlabeled target-domain samples* in addition to *labeled data from multiple source domains*, as shown in the bottom part of Figure 2. Although DG is more flexible because it does not rely on target-domain data, UDA is also highly relevant in practice because unlabeled target-domain samples are often available before deployment and can provide useful information about the target distribution. In both settings, however, achieving fairness remains challenging because sensitive attributes may be entangled with domain-specific patterns and may shift across domains.

Recent studies on fairness-aware learning have begun to address this issue by learning representations that ensure equitable outcomes across sensitive subgroups while maintaining predictive accuracy. In the DG setting, existing methods mainly focus on learning invariant representations that remain accurate across source and unseen target domains while mitigating subgroup bias. In the UDA setting, prior work has explored the use of target-domain information to align distributions and reduce unfairness caused by domain shift. For example, Quadrianto et al. (2019) proposed a fairness-aware representation learning method that reduces the dependence of predictions on sensitive attributes while aligning source and target distributions. Despite these advances, most existing approaches treat DG and UDA as separate problems (Zhang et al., 2022; Wang et al., 2020), which limits their applicability and obscures the connection between the two settings. This motivates the need for a unified fairness-aware framework that can handle both DG and UDA within a single formulation.

To bridge this gap, we substantially extend our prior work (Zhao et al., 2024), which proposed a fairness-aware framework for domain generalization. Extending DG to UDA introduces two key challenges: first, how to extract and exploit target domain specific style factors without access to target class labels; and second, how to incorporate unlabeled target-domain data into fairness-aware learning without compromising the fairness objective or requiring sensitive attributes in the target domain. To address these challenges, we propose *Fair Framework for Domain Generalization and Unsupervised Adaptation* (FFDGUA), a unified fairness-aware framework for both DG and UDA. Compared with Zhao et al. (2024), FFDGUA leverages unlabeled target-domain samples to extract target-relevant style factors and incorporates these samples into fairness-aware regularization, thereby enabling more targeted adaptation while retaining the original DG mechanism as a special case. More specifically, FFDGUA learns disentangled representations of content, style, and sensitive factors. This design promotes fairness-aware invariant learning by separating bias-related information from task-relevant content, while enabling both synthetic domain generation for DG and target-aware adaptation for UDA.

The effectiveness of FFDGUA is demonstrated through comprehensive experiments on diverse datasets, including ccMNIST, FairFace, YFCC100M-FDG, and NYSF. These datasets span multiple domains and sensitive attributes, providing a rigorous evaluation of the framework's generalization capabilities. The results demonstrate that FFDGUA achieves strong, and often state-of-the-art, performance in both fairness and accuracy, outperforming existing methods across a wide range of DG and UDA settings.

- **Unified fairness-aware framework for DG and UDA**. We propose a unified framework that addresses fairness under domain shift in both domain generalization and unsupervised domain adaptation. Unlike prior work that focuses only on DG, our formulation covers both settings within a single framework, with the original DG mechanism recovered as a special case.
- **Target-aware extension for unlabeled target domains**. To extend fairness-aware learning from DG to UDA, we introduce two key technical components: (i) a mechanism to extract style factors directly from unlabeled target-domain samples and use them to generate target-relevant augmentations, and (ii) a fairness-aware regularization strategy that incorporates unlabeled target-domain samples to better align domain-invariant representations without requiring target labels or sensitive target annotations.
- **Strong empirical performance in both DG and UDA settings**. Extensive experiments on four benchmark datasets demonstrate that the proposed framework consistently achieves a stronger balance between predictive accuracy and fairness than existing methods. Across settings with and without access to unlabeled target domain samples (UDA and DG, respectively), it yields accuracy improvements of 0.68% and 1.02%, alongside fairness gains of 6.5% and 1.75%, over the strongest respective baselines. These results highlight the benefit of unifying the two settings in a single fairness-aware framework.

The code repository is available at https://anonymous.4open.science/r/FairUDG. It will be made publicly available after the manuscript is published, allowing others to replicate our empirical results.

## 2 Related work

### 2.1 Fairness-aware domain generalization

Domain Generalization (DG) tackles the challenge of learning from multiple labeled source domains to achieve effective generalization to unseen target domains. These challenges are typically addressed by various leading techniques (Vapnik, 1999; Arjovsky et al., 2019; Sagawa et al., 2020; Yan et al., 2020; Zhang et al., 2022; Robey et al., 2021), which aim to improve the generalization ability of machine learning models across source domains characterized by distinct but potentially overlapping distributions (Volpi et al., 2021). A prevalent paradigm employs distribution alignment across sources to learn domain-invariant features (Li H. et al., 2018; Zhou et al., 2020), enabling cross-domain robustness without target domain supervision. Other approaches employ domain-aware data augmentation techniques to expand the model's exposure to potential shifts (Zhou et al., 2020). While significant progress has been made, fairness considerations in domain generalization remain underexplored. Most domain generalization research (Zhang et al., 2022; Robey et al., 2021; Blanchard et al., 2011) has focused primarily on leveraging diverse source data to identify invariant patterns. As highlighted by Blanchard et al. (2011), the central objective is to derive representations that remain robust to variations in marginal feature distributions, eliminating the need for target domain data. However, this focus has largely overlooked the challenge of ensuring fairness across domains. Bridging this gap could enhance the robustness and ethical integrity of the models deployed in practical applications. Most recently, Zhao et al. (2024) proposed an algorithmic fairness framework to tackle covariate and dependence shifts simultaneously in DG. However, the approach is heavily restricted by its reliance on labeled source data and cannot generalize to unlabeled target domains. In contrast, our proposed FFDGUA framework significantly advances this recent line of work by effectively unifying DG and UDA, utilizing a disentanglement strategy that ensures robust fairness and prediction reliability even when target labels are completely unavailable.

### 2.2 Fairness-aware unsupervised domain adaptation

Unsupervised domain adaptation (UDA) addresses the challenges of learning from multiple labeled source domains and unlabeled target domains, with the goal of generalizing effectively to target domains. Conventional UDA approaches (Ganin et al., 2016; Hoffman et al., 2018; Peng et al., 2019) focus on mitigating domain shifts by aligning distributions between source and target domains, often through adversarial learning or domain-invariant representation learning. These approaches assume that combining information from multiple source domains can lead to more robust feature extraction, improving the performance on the target domain (Sun and Saenko, 2016; Zhao et al., 2018). Recent advancements have incorporated fairness into UDA to address ethical concerns arising from domain-specific biases. Several studies have aimed to disentangle sensitive attributes from domain-invariant features to ensure equitable model behavior across different groups (Wang et al., 2020; Quadrianto et al., 2019). However, these approaches often rely on explicit sensitive attribute labels in both source and target domains, limiting their applicability in real-world settings where such labels are often unavailable.

### 2.3 Distribution shifts in DG and UDA

**Figure 3:**
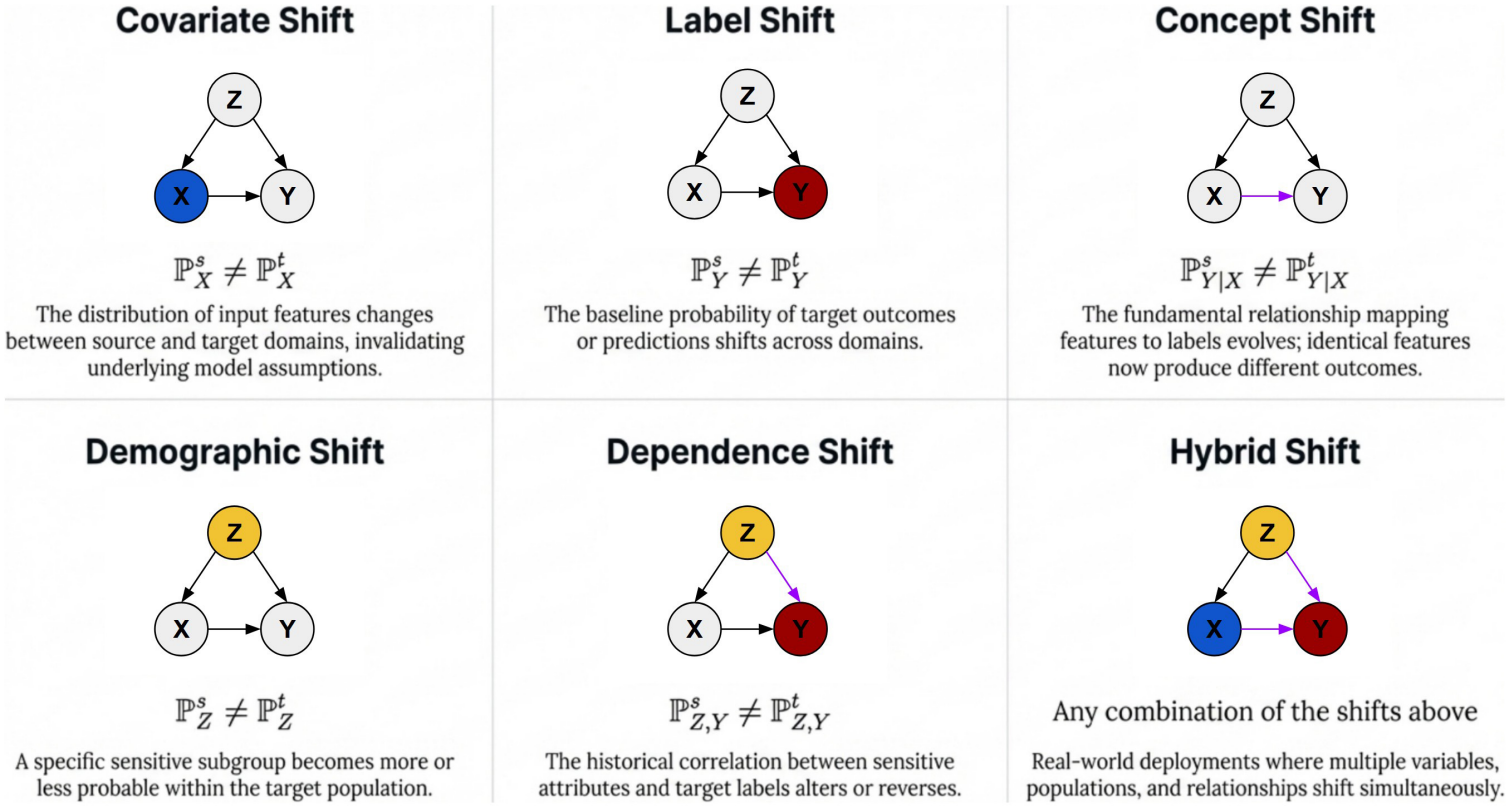
A taxonomy of distribution shifts in real-world domain generalization, where *X, Y, Z* denote features, class labels, and sensitive attributes. While practical deployments may encounter a complex multitude of shifts, such as label, concept, and demographic shifts, our FFDGUA framework explicitly bounds its scope to Covariate Shift (style variations in input features) and Dependence Shift (variations in the correlation between sensitive attributes and target labels).

Real-world DG deployments may encounter a diverse range of distribution shifts that can substantially degrade model performance. As demonstrated in Figure 3 (Shao et al., 2024), these shifts include covariate, label, concept, demographic, dependence, and hybrid shifts. Existing methods have primarily focused on *covariate shift*, where the input distribution changes while the labeling mechanism remains stable, often through data augmentation or invariance-inducing strategies (Zhang et al., 2022; Robey et al., 2021). In contrast, *label shift* refers to changes in class prior probabilities and is commonly addressed by methods such as importance-weighted adversarial learning (Tachet des Combes et al., 2020) or label disambiguation techniques (Xiao et al., 2026). *Concept shift*, in which the underlying feature-to-label relationship changes, remains particularly challenging and has been studied through approaches based on ridge regression (Nguyen et al., 2024) or internal distribution consolidation (Rostami and Galstyan, 2023). In fairness-aware learning, *demographic shift*, which changes subgroup proportions, has been addressed using density-matching frameworks (Pham et al., 2023; Giguere et al., 2022). *Dependence shift*, which alters the correlation between sensitive attributes and target labels, has increasingly been studied through content–style disentanglement and transformation-based synthetic domain generation (Zhao et al., 2024). Finally, *hybrid shifts*, which involve multiple shift types simultaneously, generally require more flexible probabilistic inference mechanisms (An et al., 2022).

### 2.4 Style variations in DG and UDA

Existing DG literature can also be viewed through the lens of how it models style variation across domains. One common setting assumes that source and target domains share some underlying basic style variations, implying an overlapping latent space in which the fundamental structures of different domains remain partially aligned (e.g., images collected under different lighting conditions, camera sensors, backgrounds, or weather conditions, while preserving the same semantic objects and labeling rules). Methods in this setting typically aim to suppress domain-specific noise and recover stable factors that are shared across related tasks (Arjovsky et al., 2019; Zhang et al., 2022; Robey et al., 2021; Zhao et al., 2024). A second, more challenging setting considers extreme distribution shifts, where the target domain exhibits styles or feature spaces that are largely unseen during training (e.g., training on natural photographs but testing on sketches, cartoons, or heavily corrupted images whose appearance departs substantially from the source domains). Methods for this regime often adopt an expansion strategy, seeking robustness by exposing the model to synthesized or adversarially generated variations (Volpi et al., 2018; Zhou et al., 2021; Li et al., 2019).

### 2.5 Scope of our work

*(a) Distribution shifts*. Although real-world deployments may involve label, concept, demographic, and other complex shifts, modeling all such variations within a single framework would require substantially stronger assumptions and a much broader problem formulation. We therefore deliberately restrict our focus to *style-induced covariate shift* and *dependence shift*, which are the two shift types explicitly modeled by our framework. This choice is aligned with the design of our transformation model, which assumes that cross-domain variation arises from changes in input style and in the relationship between sensitive attributes and labels. Under this bounded scope, the model disentangles domain-invariant content from domain-specific style factors and fairness-relevant sensitive correlations, and then synthesizes augmented domains by perturbing these factors. Building on prior DG methods that rely on shared structural assumptions, it allows our framework to simulate unseen environments by sampling from a continuous prior over shared basic variations, rather than attempting to model the full complexity of jointly occurring shift types. *(b) Style variations*. Our method is designed for the setting in which source and target domains share some basic style variations. Following prior DG works that make similar shared-structure assumptions (Zhang et al., 2022; Robey et al., 2021), our transformation model samples perturbations from a continuous prior over these shared latent variations. Accordingly, the intended generalization regime is not arbitrary domain shift in the strongest possible sense, but rather unseen domains that remain compatible with the shared latent structure inferred from the source domains.

### 2.6 Our unified framework

Our framework enhances fairness-aware disentanglement techniques by reducing reliance on explicit sensitive attribute labels, thereby improving applicability in unsupervised adaptation settings. It ensures that the model learns fairness-preserving representations that are domain-invariant while being generalizable to the target domain. By learning fairness-aware parameters, our method addresses the limitations of prior works that struggle with fairness-aware generalization in the target domains.

## 3 Preliminaries

### 3.1 Notations

Let $X\subseteq {\mathbb{R}}^{d}$ represent the feature space, Z={-1,1} denote the sensitive attribute space, and Y={0,1} be the binary label space for classification. Consider three latent spaces: $C\subseteq {\mathbb{R}}^{c}$ (content), $A\subseteq {\mathbb{R}}^{a}$ (sensitive), and $S\subseteq {\mathbb{R}}^{s}$ (style). Let *X, Z, Y, C, A, S* denote random variables taking values in X,Z,Y,C,A,S respectively, with corresponding realizations **x**, *z, y*, **c**, **a**, **s**. Let ℰ represent all possible domains and ${ℰ}^{s}\subset ℰ$ denote the source domains. A domain e∈ℰ is characterized by its joint distribution ${\mathbb{P}}_{XZY}^{e}=\mathbb{P}\left(X^{e},Z^{e},Y^{e}\right):X\times Z\times Y\to \left[0,1\right]$. A classifier *f* from the hypothesis class ℱ is defined as f∈ℱ:X→Y. The superscripts indicate domain membership (e.g., **x**^*s*^ denotes a sample from domain *s*), while the subscripts specify encoder indices (e.g., $E_{c}\left(x^{s}\right)$ represents a content encoder *E*_*c*_ processing a domain *s* sample). Important notations are listed in Table 1.

**Table 1:** Important notations and corresponding descriptions.

Notations	Descriptions
X,Z,Y,Θ	input feature space, sensitive space, output space, and parameter space
C,S,A	latent space for content factors, style factors, and sensitive factors
**c, s, a**	content factor, style factor, and sensitive factor
*d*[·]	distance metric over outputs
D	data set
$D^{s},D^{t}$	available samples in source domains and target domain
**x**, *z, y*	data features, sensitive attribute, and class label
$f,ℱ,\hat{f}$	classifier, classifier space and its ξ-parameterization
ŷ	predicted class label
*g*(, )	fairness function
*p*_1_	empirical estimate of the proportion of samples in the group *z* = 1
e,ℰ	domain and set of domains
ℬ	sampled data batch
*T*	domain transformation model
ℒ	loss function
δ	empirical relaxed constraint
ẑ	sensitive attributes predicted by *h*
η_*p*_, η_*d*_	primal and dual learning rate
λ, γ	dual variable and empirical constant

### 3.2 Group fairness

When learning a fair classifier f∈ℱ that aims to achieve statistical parity across distinct sensitive subgroups, the fairness criteria require the independence between the sensitive random variables *Z* and the model's predicted outcome *f*(*X*) (Dwork et al., 2011). The problem of preventing group unfairness can be formulated as a constraint. This constraint reduces bias by ensuring that *f*(*X*) is aligned with the ground truth *Y*, thereby promoting fairness in outcomes.

** Definition 1 (Group Fairness Notion (Wu et al., 2019; Lohaus et al., 2020))**. Given a dataset $D={\left\{\left(x_{i},z_{i},y_{i}\right)\right\}}_{i=1}^{\left|D\right|}$ sampled *i.i.d*. from ℙ_*XZY*_, a classifier f∈ℱ:X→Y is considered fair when the prediction Ŷ = *f*(*X*) is independent of the sensitive random variable *Z*. To eliminate the indicator function and relax the exact values, a linearly approximated form of the difference between sensitive subgroups is defined as


$$\begin{array}{l} \rho (\hat{Y},Z)=\left|E_{{\mathbb{P}}_{XZY}}g(\hat{Y},Z)\right|\;where\;g(\hat{Y},Z)=\frac{1}{p_{1}\left(1-p_{1}\right)}\left(\frac{Z+1}{2}-p_{1}\right)\hat{Y} \end{array}$$ (1)

Here, *p*_1_ = ℙ(*Z* = 1) and 1−*p*_1_ = ℙ(*Z* = −1) represent the proportion of samples in the subgroups *Z* = 1 and *Z* = −1, respectively. Thus Equation 1 corresponds to the difference in demographic parity, where its expectation is over *XZ*.

Intuitively, Definition 1 states that a model is *fair across domains* if changing the sensitive attribute (e.g., race or gender) does not alter the model's predictions after accounting for how different domains transform the data. In other words, even though each domain may distort or shift the inputs in its own way, the model should behave the same for individuals who are identical in all respects except for the sensitive attribute. This captures the idea that fairness must hold not only within each domain but also across domains, ensuring that the model's decisions remain stable and unbiased even when the environment or data distribution changes.

This definition can also be extended to the difference in equalized opportunity when *p*_1_ = *P*(*Z* = 1, *Y* = 1) (Lohaus et al., 2020). In this paper, we present results under demographic parity, while the framework can be extended to multi-class, multi-sensitive attributes, and other fairness notions. Strictly speaking, a classifier *f* is fair across subgroups if it satisfies ρ(Ŷ, *Z*) = 0.

### 3.3 Problem setting

Given a dataset $D={\left\{D^{e}\right\}}_{e=1}^{\left|ℰ\right|}$, where each $D^{e}={\left\{\left(x_{i}^{e},z_{i}^{e},y_{i}^{e}\right)\right\}}_{i=1}^{\left|D^{e}\right|}$ is *i.i.d*. sampled from a domain ${\mathbb{P}}_{XZY}^{e}$ and e∈ℰ, we consider multiple source domains ${\left\{{\mathbb{P}}_{XZY}^{s}\right\}}_{s=1}^{\left|{ℰ}^{s}\right|}$ and a distinct target domain ${\mathbb{P}}_{XZY}^{t}$, where *t*≠*s* for all $s\in {ℰ}^{s}\subset ℰ$ and $t\in ℰ\backslash {ℰ}^{s}$. Given samples ${\left\{D^{e}\right\}}_{e=1}^{\left|{ℰ}^{s}\right|}$ from finite domains, the objective of fairness-aware domain generalization is to learn a classifier f∈ℱ that generalizes well to the target domain.

A key challenge is determining how closely the data distributions in the target domain align with those in observed source domains. While various types of distribution shifts exist, we attribute them solely to covariate and dependence shifts in this study, since they are the two major types of distribution shifts occurring between source and target domains (Roh et al., 2023).

**Definition 2** (Covariate Shift (Robey et al., 2021) and Dependence Shift (Roh et al., 2023)). Covariate shift occurs when differences in domain distributions are due to variations in the marginal distributions of input features, expressed as ${\mathbb{P}}_{X}^{s}\neq {\mathbb{P}}_{X}^{t},\forall s$. Conversely, dependence shift arises when changes in the domain result from modifications in the joint distribution of *Y* and *Z*, represented as ${\mathbb{P}}_{YZ}^{s}\neq {\mathbb{P}}_{YZ}^{t},\forall s$. This occurs either when ${\mathbb{P}}_{Y|Z}^{s}\neq {\mathbb{P}}_{Y|Z}^{t}$ while ${\mathbb{P}}_{Z}^{s}={\mathbb{P}}_{Z}^{t}$, or when ${\mathbb{P}}_{Z|Y}^{s}\neq {\mathbb{P}}_{Z|Y}^{t}$ while ${\mathbb{P}}_{Y}^{s}={\mathbb{P}}_{Y}^{t}$.

Definition 2 provides an intuitive way to understand how the model separates domain-specific information from the features relevant to prediction and fairness. In simple terms, it states that the transformation function should isolate the factors that vary across domains while leaving the underlying semantic content intact. This means that when data from different domains are mapped through the transformation, their domain-dependent variations are removed, allowing the model to make predictions based on the true underlying signal rather than on spurious domain artifacts. As a result, the model becomes more robust and fair when encountering new or shifted domains.

## 4 Methodology

### 4.1 Overview of the FFDGUA framework

Before detailing the individual components, we present a holistic overview of the FFDGUA workflow. The framework operates in two interconnected stages to unify Domain Generalization (DG) and Unsupervised Domain Adaptation (UDA).

**Figure 4:**
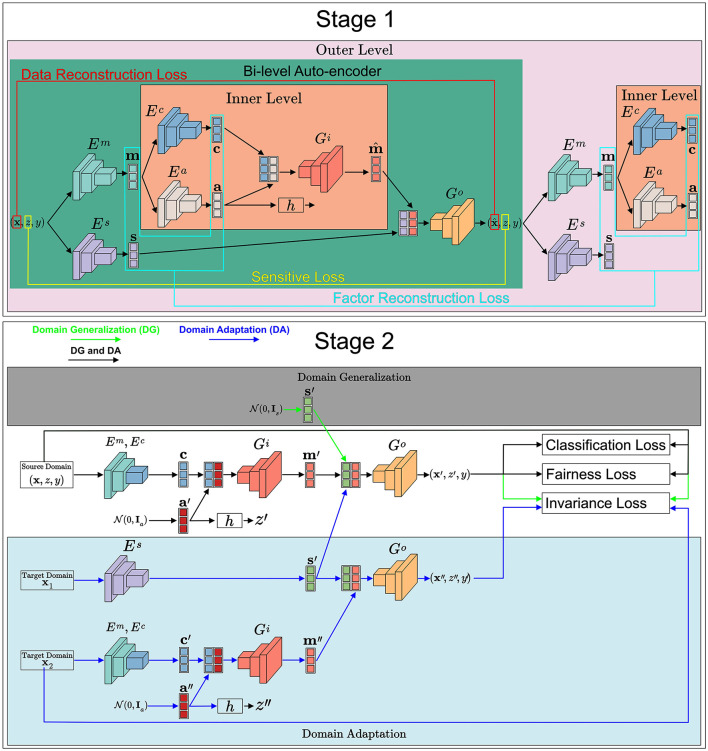
An overview of our FFDGUA framework. The learned transformation model from Stage 1 generates augmented samples to train a robust classifier. For DG, it synthesizes arbitrary unseen domains by sampling style factors from a prior Gaussian distribution. For UDA, it explicitly extracts exact style factors from unlabeled target domain samples. Finally, the framework optimizes the classifier using a joint objective of losses.

Stage 1: Disentanglement and Transformation: The framework first trains a bi-level auto-encoder (the top part of Figure 4) to decouple input data into three distinct latent spaces: domain-invariant content, domain-specific style, and sensitive factors.

Stage 2: Fairness-Aware Generation and Classification (the bottom part of Figure 4): Using the transformation model from Stage 1, FFDGUA dynamically generates augmented samples. For DG, it synthesizes arbitrary unseen domains by randomly sampling style and sensitive factors. For UDA, it encodes style factors directly from unlabeled target domain samples. Finally, a classifier is trained on both the original and augmented data, guided by classification, domain-invariance, and fairness losses to ensure robust and fair predictions.

### 4.2 Underlying transformation models

Recent advances in domain generalization utilize transformation models to address domain shifts via latent space manipulations. Robey et al. (2021) proposed a model that simulates environmental factors and enforces prediction invariance through relaxed regularization constraints using image-to-image translation. Zhang et al. (2022) designed a framework combining encoders and a decoder with latent factor swapping and adversarial training to simulate unseen variations. Zhao et al. (2023) disentangle semantic, style, and sensitive factors, enabling fairness-aware augmentation that improves robustness to both covariate and dependence shifts.

Inspired by existing efforts in domain generalization (Robey et al., 2021; Zhang et al., 2022; Huang et al., 2018), distribution shifts can be represented by an underlying transformation model *T*, characterizing generalization tasks across domains. The motivation for utilizing *T* is to enhance the robustness and adaptability of the classifier *f* in the target domains. By learning a transformation model, the objective is twofold: (1) to enable the extraction of domain-invariant data representations (factors) from input data by disentangling domain-specific factors, and (2) to generate augmented data in new domains or the target domain by perturbing existing samples with different variations. This augmentation process diversifies the source data, thereby improving the model's ability to generalize effectively to target domains.

**Figure 5:**
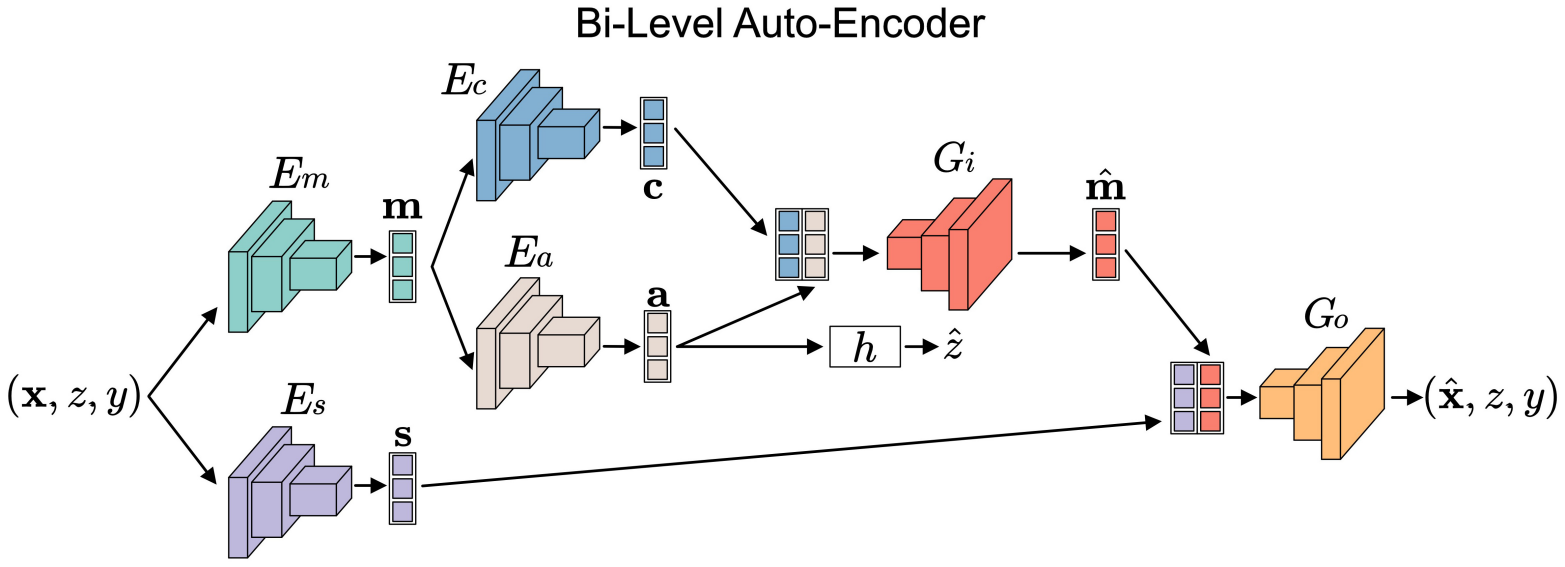
The bi-level auto-encoder of our transformation framework. To disentangle each datapoint to latent content, style, and sensitive factors within corresponding latent spaces, stage 1 of the proposed framework aims to train a set of encoders, decoders, and discriminators with a two-level architecture. For simplicity, discriminators are omitted.

We consider a bi-level auto-encoder for our transformation model as shown in Figure 5. One goal of the transformation model *T* = {*E, G*} is to disentangle an input sample from source domains into three factors in latent spaces by learning a set of encoders *E* = {*E*_*m*_, *E*_*c*_, *E*_*a*_, *E*_*s*_} and a set of decoders *G* = {*G*_*i*_, *G*_*o*_}. $E_{m}:X\to ℳ$, $E_{c}:ℳ\to C$, $E_{a}:ℳ\to A$, $E_{s}:X\to S$ represent the semantic (*m*), content (*c*), sensitive (*a*), and style (*s*) encoders, respectively. $G_{i}:C\times A\to ℳ$ and $G_{o}:ℳ\times S\to X$ represent the inner and outer level decoders, respectively.

**Assumption 1** (Latent Spaces). Given a dataset $D^{e}={\left\{\left(x_{i}^{e},z_{i}^{e},y_{i}^{e}\right)\right\}}_{i=1}^{\left|D^{e}\right|}$ drawn *i.i.d*. from domain e∈ℰ with distribution ${\mathbb{P}}_{XZY}^{e}$, we posit that each instance $x_{i}^{e}$ arises from three latent components: (a) A content factor c∈C; (b) A sensitive factor a∈A ; (c) A style factor **s**^*e*^ specific to domain *e*. The content and sensitive factors in C and A remain invariant across domains, while style factors **s**^*e*^ vary between domains. Each domain ${\mathbb{P}}_{XZY}^{e}$ is parameterized as *e*: = (**s**^*e*^, ρ^*e*^), where ρ^*e*^ = ρ(*Y*^*e*^, *Z*^*e*^)^1^ is the difference between sensitive subgroups in *e*. The pair (**s**^*e*^, ρ^*e*^) uniquely identifies domain ${\mathbb{P}}_{XZY}^{e}$.

Assumption 1 informally states that the observed data in each domain can be explained by a shared underlying structure together with domain-specific variations. This means that while different domains may introduce their own shifts or distortions, they all originate from the same latent semantic factors. By assuming this shared structure, the model can meaningfully disentangle domain-invariant information from domain-dependent noise, enabling more stable prediction and improved fairness across domains. In practice, this assumption ensures that learning from multiple domains is feasible because they are related through a common generative process.

Note that Assumption 1 is closely related to the assumptions made in Zhang et al. (2022); Robey et al. (2021); Huang et al. (2018); Liu et al. (2017). In our paper, with a focus on group fairness, we extend the assumptions of existing works by introducing three latent factors. Under Assumption 1, if two instances $\left(x^{e_{i}},z^{e_{i}},y\right)$ and $\left(x^{e_{j}},z^{e_{j}},y\right)$, where $e_{i},e_{j}\in ℰ$ and *i*≠*j*, share the same class label, then the latter instance can be reconstructed from the former as $\left(x^{e_{j}},z^{e_{j}}\right)=T\left(x^{e_{i}},z^{e_{i}},e_{j}\right)$. In detail, $c^{e_{i}}=E_{c}\left(E_{m}\left(x^{e_{i}}\right)\right)$, $x^{e_{j}}=G_{o}\left(G_{i}\left(c^{e_{i}},a^{e_{j}}\right),s^{e_{j}}\right)$ and $z^{e_{j}}=h\left(a^{e_{j}}\right)$, where $a^{e_{j}}$ and $s^{e_{j}}$ are the sensitive and style factors from domain *e*_*j*_.

Specifically, in the outer level, an instance is first encoded to a semantic factor m∈ℳ and a style factor s∈S through the corresponding encoders *E*_*m*_ and *E*_*s*_, respectively. In the inner level, the semantic factor **m** is further encoded to a content factor c∈C and a sensitive factor a∈A, through encoders *E*_*c*_ and *E*_*a*_.

To improve the effectiveness of the transformation model *T*, our overall learning loss for encoders and decoders comprises three key components: a bidirectional reconstruction loss, a sensitiveness loss, and an adversarial loss.

**Data reconstruction loss** promotes learning reconstruction in the direction of data → latent → data. Specifically, a data sample **x**^*e*^ from ${\mathbb{P}}_{X}^{e}$, for all e∈ℰ, is required to be reconstructed using its encoded factors (Equation 2).


$$\begin{array}{l} {ℒ}_{\text{recon }}^{\text{data }}=E_{x^{e}\sim {\mathbb{P}}_{X}^{e}}\left[{\left\|G_{o}\left(\hat{m},E_{s}\left(x^{e}\right)\right)-x^{e}\right\|}_{1}\right]+E_{m\sim {\mathbb{P}}_{M}}[\|G_{i}\left(E_{c}(m),E_{a}(m)\right)\\ -m{\|}_{1}] \end{array}$$ (2)

where $\hat{m}=G_{i}\left(c,a\right)=G_{i}E_{c}\left(E_{m}\left(x^{e}\right)\right),E_{a}\left(E_{m}\left(x^{e}\right)\right)$; ℙ_*M*_ is given by $m=E_{m}\left(x^{e}\right)$.

#### 4.2.1 Factor reconstruction loss

Given latent factors **c**, **a**, and **s**^*e*^ encoded from a sample **x**^*e*^, they are encouraged to be reconstructed using latent factors randomly sampled from the prior Gaussian distributions (Equation 3).


$$\begin{array}{l} {ℒ}_{recon\;}^{factor\;}=E_{c∼{\mathbb{P}}_{C},a∼N\left(0,I_{a}\right)}\left[E_{c}\left(G_{i}(c,a)\right)-c_{1}\right]\\ +E_{c∼{\mathbb{P}}_{C},a∼N\left(0,I_{a}\right)}\left[E_{a}\left(G_{i}(c,a)\right)-a_{1}\right]\\ +E_{m∼{\mathbb{P}}_{M},s^{e}∼N\left(0,I_{s}\right)}\left[E_{s}\left(G_{o}(m,s)\right)-s_{1}\right]\\ +E_{m∼{\mathbb{P}}_{M},s^{e}∼N\left(0,I_{s}\right)}\left[E_{m}\left(G_{o}(m,s)\right)-m_{1}\right]\\ +E_{c∼{\mathbb{P}}_{C},s^{e}∼N\left(0,I_{s}\right),a∼N\left(0,I_{a}\right)}\left[E_{s}\left(G_{o}\left(G_{i}(c,a),s\right)\right)-s_{1}\right] \end{array}$$ (3)

where ℙ_*C*_ and ℙ_*M*_ are given by $c=E_{c}\left(E_{m}\left(x^{e}\right)\right)$ and $m=E_{m}\left(x^{e}\right)$. $a=E_{a}\left(E_{m}\left(x^{e}\right)\right)$, and $s=E_{s}\left(x^{e}\right)$.

The reconstruction loss (Equation 4) is defined as follows:


$$\begin{array}{l} {ℒ}_{recon}={ℒ}_{recon}^{data}+{ℒ}_{recon}^{factor} \end{array}$$ (4)

#### 4.2.2 Sensitiveness loss

Since a sensitive factor is causally dependent on the sensitive attribute of the source domain data (**x**^*s*^, *z*^*s*^, *y*^*s*^), a simple classifier h:A→Z is learned. This classifier is further used to label the sensitive attribute in augmented data when training *f* (Equation 5).


$$\begin{array}{l} {ℒ}_{sens}=CrossEntropy\left(z^{s},h\left(E_{a}\left(x^{s}\right)\right)\right) \end{array}$$ (5)

#### 4.2.3 Adversarial loss

Inspired by the success of Generative Adversarial Networks (GANs) (Goodfellow et al., 2020) in data augmentation for evaluating disentanglement in latent spaces, we leverage GANs to align the distribution of reconstructed data with the original data distribution. As discussed in Huang et al. (2018), data synthesized by our encoders and decoders should be indistinguishable from real data within the same domain (Equation 6).


$$\begin{array}{l} {ℒ}_{adv}=E_{c∼{\mathbb{P}}_{C},s^{e}∼N\left(0,I_{s}\right),a∼N\left(0,I_{a}\right)}\left[\log\left(1-D_{o}\left(G_{o}\left(\hat{m},s^{e}\right)\right)\right)\right]\\ +E_{c∼{\mathbb{P}}_{C},a∼N\left(0,I_{a}\right)}\left[\log\left(1-D_{i}\left(G_{i}(c,a)\right)\right)\right]\\ +E_{x^{e}∼{\mathbb{P}}_{X}^{s}}\left[\log D_{o}\left(x^{e}\right)\right]+E_{m∼{\mathbb{P}}_{M}}\left[\log D_{i}(m)\right] \end{array}$$ (6)

where $D_{o}:X\to \mathbb{R}$ and $D_{i}:ℳ\to \mathbb{R}$ are the discriminators for the outer and inner levels, respectively.

#### 4.2.4 Total loss

We simultaneously train the encoders, decoders, and discriminators to optimize the overall objective (Equation 7):


$$\begin{array}{l} \underset{E,G}{\min}\underset{D}{\max}{ℒ}_{total\;}(E,G,D)={ℒ}_{recon\;}+\beta_{z}{ℒ}_{cls}^{z}+\beta_{g}{ℒ}_{GAN} \end{array}$$ (7)

where *D* = {*D*_*i*_, *D*_*o*_}. The parameters β_*z*_, β_*g*_>0 control the relative importance of each loss term in the formula.

### 4.3 Fair disentangled domain generalization

Given a transformation model *T*, we aim to train a fairness-aware invariant classifier *f* across multiple domains under the following assumption.

**Assumption 2** (Fairness-aware Domain Shift). We assume that variations across domains are driven by covariate and dependence shifts. Consequently, the conditional distribution ${\mathbb{P}}_{Y|XZ}^{e}$ remains invariant across all domains, i.e., ∀e∈ℰ. Given a transformation function *T*, this assumption implies that ${\mathbb{P}}_{Y|XZ}^{e_{i}}={\mathbb{P}}_{Y|XZ}^{e_{j}}$ for all domain pairs $e_{i},e_{j}\in ℰ,i\neq j$, where the transformation follows $\left(X^{e_{j}},Z^{e_{j}}\right)=T\left(X^{e_{i}},Z^{e_{i}},e_{j}\right)$.

Assumption 2 informally states that the transformation applied to each domain preserves the essential semantic information needed for prediction and fairness. In other words, while domains may differ in their appearance or distribution, the transformation does not distort the core content of the data that the model relies on. This ensures that when samples from different domains are mapped into a shared space, their meaningful structure is retained, allowing the model to compare them fairly and learn domain-invariant representations. This assumption is crucial for guaranteeing that the learned features remain informative while still removing harmful domain-specific biases.

In Assumption 2, the transformation model *T* captures the domain shift by mapping the marginal distributions ${\mathbb{P}}_{X}^{e_{i}}$ and $\rho \left(Y^{e_{i}},Z^{e_{i}}\right)$ in domain *e*_*i*_ to the corresponding distributions ${\mathbb{P}}_{X}^{e_{j}}$ and $\rho \left(Y^{e_{j}},Z^{e_{j}}\right)$ in a different domain *e*_*j*_, sampled from ${\mathbb{P}}_{XZY}^{e_{j}}$. Building upon this, we introduce a refined definition of fairness-aware invariance. This definition is aligned with the variations encapsulated by *T* and ensures compliance with the group fairness constraint as specified in Definition 1.

**Definition 3** (Fairness-aware T-Invariance). Given a domain transformation model *T*, a classifier is fairness-aware and domain invariant if (Equation 8):


$$\begin{array}{l} f\left(x^{e_{i}}\right)=f\left(x^{e_{j}}\right),\;\;and\;\;\rho \left(f\left(X^{e_{i}}\right),Z^{e_{i}}\right)=\rho \left(f\left(X^{e_{j}}\right),Z^{e_{j}}\right)=0 \end{array}$$ (8)

is satisfied with high probability, where $\left(x^{e_{j}},z^{e_{j}}\right)=T\left(x^{e_{i}},z^{e_{i}},e_{j}\right)$, $x^{e_{i}}∼{\mathbb{P}}_{X}^{e_{i}},x^{e_{j}}∼{\mathbb{P}}_{X}^{e_{j}}$.

Definition 3 informally states that the model's representation should capture the true semantic content of the data while filtering out variations that arise from domain shifts or sensitive attributes. In essence, it requires that the learned features remain stable and meaningful even when the input comes from different domains or undergoes different transformations. This ensures that the predictor relies on information that is genuinely relevant to the task, rather than on spurious cues introduced by domain-specific artifacts. As a result, the model becomes more robust, interpretable, and fair when making decisions across heterogeneous environments.

Definition 3 is designed for directly enforcing invariance on the predictions generated by *f*. It ensures that a prediction remains stable across different data realizations induced by *T*, while maintaining considerations of group fairness.

**Problem 1** (Fairness-aware Domain Generalization with T-Invariance). Given the definitions and assumptions outlined in Definition 3 and Assumption 2 and a loss function ℓ:Y×Y→ℝ, we define the fairness-aware domain generalization with T-invariance problem as follows:


$$\begin{array}{l} P^{⋆}≜\underset{f\in ℱ}{\min}R(f)≜E_{{\mathbb{P}}_{XZY}^{s_{i}}}\ell \left(f\left(X^{s_{i}}\right),Y^{s_{i}}\right)\\ \;s.t.\;f\left(X^{e_{j}}\right)=f\left(X^{e_{k}}\right),\rho \left(f\left(X^{e_{k}}\right),Z^{e_{k}}\right)=\rho \left(f\left(X^{e_{j}}\right),Z^{e_{j}}\right)=0 \end{array}$$ (9)

where $\forall s_{i}\in {ℰ}^{s},e_{j},e_{k}\in ℰ,j\neq k,\left(X^{e_{j}},Z^{e_{j}}\right)\in D^{e_{j}}$, and $\left(X^{e_{k}},Z^{e_{k}}\right)=T\left(X^{e_{j}},Z^{e_{j}},e_{k}\right)$. The accessibility of $D^{t}$ (unlabeled target domain samples) distinguishes between the two scenarios:

- Fairness-aware domain generalization: $|D^{t}|=0$, where there is no available samples in the target domain. In this scenario, the problem equals to:

$$\begin{array}{l} P^{⋆}≜\underset{f\in ℱ}{\min}R(f)≜E_{{\mathbb{P}}_{XZY}^{s_{i}}}\ell \left(f\left(X^{s_{i}}\right),Y^{s_{i}}\right)\\ \;s.t.\;f\left(X^{s_{j}}\right)=f\left(X^{s_{i}}\right),\rho \left(f\left(X^{s_{j}}\right),Z^{s_{j}}\right)=\rho \left(f\left(X^{s_{i}}\right),Z^{s_{i}}\right)=0 \end{array}$$ (10)

where $\forall s_{i},s_{j}\in {ℰ}^{s},i\neq j$, and $\left(X^{s_{j}},Z^{s_{j}}\right)=T\left(X^{s_{i}},Z^{s_{i}},s_{j}\right)$.

- Fairness-aware unsupervised domain adaptation: $D^{t}={\left\{x_{i}^{t}\right\}}_{i=1}^{\left|D^{t}\right|}$ and $|D^{t}|>0$, where the unlabeled samples are assumed to be drawn i.i.d. from the marginal target distribution. Since only unlabeled target domain samples are available, $Z^{e_{j}}\in Z$ is randomly sampled.

Equations 9, 10 informally asks the model to learn a predictor that remains both accurate and fair while generalizing to unseen domains. In fairness-aware domain generalization, the model learns from fully labeled data in multiple source domains and must generalize fairly to target domains that provide no samples during training, requiring representations that stay stable and unbiased under domain shifts. In contrast, fairness-aware unsupervised domain adaptation assumes that, in addition to labeled source data, a set of unlabeled target-domain samples is available. These unlabeled samples reveal how the new domain deviates from the sources, enabling better alignment of representations and improved fairness. Together, the two settings capture realistic deployment scenarios: one where no target-domain data can be accessed in advance, and another where limited but unlabeled target information helps guide fair generalization.

Similar to Robey et al. (2021), problem 1 does not constitute a composite optimization problem. Furthermore, obtaining domain labels is often costly or even infeasible, particularly due to privacy concerns. Optimizing problem 1 presents several challenges: (1) The strict equality constraints in problem 1 are difficult to enforce in practice. (2) Enforcing constraints within deep networks is inherently challenging due to their non-convex nature. Simply converting these constraints into regularization terms does not guarantee compliance with the original constrained optimization problem. (3) The incomplete availability of data across all domains restricts the ability to enforce fairness-aware *T*-invariance, further complicating the estimation of *R*(*f*).

Given these challenges, we propose a tractable approach to approximately solve problem 1. To mitigate the first challenge, we introduce a relaxation of the constraints in problem 1 (Equations 11–13):


$$\begin{array}{l} P^{⋆}\left(\gamma_{1},\gamma_{2}\right)≜\underset{f\in ℱ}{\min}R(f)\;s.t.\;\delta^{e_{j},e_{k}}(f)\leq \gamma_{1},\rho^{e_{j}}(f)\leq \frac{\gamma_{2}}{2},\;\text{and}\;\rho^{e_{k}}(f)\leq \frac{\gamma_{2}}{2} \end{array}$$ (11)

where


$$\begin{array}{ll} \delta^{e_{j},e_{k}}\left(f\right) & ≜E_{{\mathbb{P}}_{XZ}^{e_{j}}}df\left(X^{e_{j}}\right),f\left(X^{e_{k}}=T\left(X^{e_{j}},Z^{e_{j}},e_{k}\right)\right), \end{array}$$ (12)

$$\begin{array}{ll} \rho^{e_{j}}\left(f\right) & ≜\rho \left(f\left(X^{e_{j}}\right),Z^{e_{j}}\right),\;\rho^{e_{k}}\left(f\right)≜\rho \left(f\left(X^{e_{k}}\right),Z^{e_{k}}\right) \end{array}$$ (13)

where $e_{j},e_{k}\in ℰ,j\neq k$, and *d*[·, ·] represents a distance metric, such as KL-divergence. Here, γ_1_, γ_2_>0 are constants that control the degree of relaxation. When γ_1_ = γ_2_ = 0, Equations 9, 11 become equivalent.

We formulate the empirical dual problem based on data sampled from ℰ (Equation 14):


$$\begin{array}{l} D_{\xi ,ℰ}^{⋆}\left(\gamma_{1},\gamma_{2}\right)≜\underset{\lambda_{1}\left(e_{j},e_{k}\right),\lambda_{2}\left(e_{i},e_{l}\right)}{\max}\underset{\theta \in \text{Θ}}{\min}\hat{R}(\theta )\\ +\frac{1}{\left|ℰ\right|}\sum_{e_{j},e_{k}\in ℰ}[\lambda_{1}\left(e_{j},e_{k}\right)\left({\hat{\delta }}^{e_{j},e_{k}}(\theta )-\gamma_{1}\right)\\ +\lambda_{2}\left(e_{j},e_{k}\right)\left({\hat{\rho }}^{e_{j}}(\theta )+{\hat{\rho }}^{e_{k}}(\theta )-\gamma_{2}\right)] \end{array}$$ (14)

where $\xi =E_{{\mathbb{P}}_{X}}||f\left(x\right)-\hat{f}\left(x,\theta \right)|{|}_{\infty }>0$ represents a constant that provides an upper bound on the discrepancy between the function *f* and its parameterized approximation $\hat{f}:X\times \Theta \to \mathbb{R}$. The terms λ_1_(*e*_*j*_, *e*_*k*_), λ_2_(*e*_*j*_, *e*_*k*_)>0 denote dual variables. Additionally, $\hat{R}\left(\theta \right)$, ${\hat{\delta }}^{e_{j},e_{k}}\left(\theta \right)$, ${\hat{\rho }}^{e_{j}}\left(\theta \right)$, and ${\hat{\rho }}^{e_{k}}\left(\theta \right)$ represent the empirical approximations of $R\left(\hat{f}\left(\cdot ,\theta \right)\right)$, $\delta^{e_{j},e_{k}}\left(\hat{f}\left(\cdot ,\theta \right)\right)$, $\rho^{e_{j}}\left(\hat{f}\left(\cdot ,\theta \right)\right)$, and $\rho^{e_{k}}\left(\hat{f}\left(\cdot ,\theta \right)\right)$, respectively.

### 4.4 The FFDGUA algorithm

FFDGUA is guided by three complementary losses: classification loss, invariance loss, and fairness loss. These losses collectively ensure that the model achieves high prediction performance while maintaining fairness and domain-invariance in target domains.

#### 4.4.1 Classification loss

The classification loss is the cornerstone of the framework, responsible for optimizing the prediction accuracy of the classifier *f*. It measures the discrepancy between the predicted labels and the ground-truth labels for samples in the source domains. Given a data batch $ℬ={\left\{\left(x_{i},y_{i},z_{i}\right)\right\}}_{i=1}^{\left|ℬ\right|}$ and a classifier *f* parameterized by ***θ***, the classification loss ${ℒ}_{cls}\left(\theta ,ℬ\right)$ is expressed as (Equation 15):


$$\begin{array}{l} {ℒ}_{cls}\left(\theta ,ℬ\right)=\frac{1}{\left|ℬ\right|}\overset{\left|ℬ\right|}{\underset{i=1}{\sum }}\ell \left(y_{i},f\left(x_{i},\theta \right)\right) \end{array}$$ (15)

where the distance metric ℓ(·) is defined using cross-entropy.

#### 4.4.2 Invariance loss

The invariance loss enforces consistency between distributions of the latent representations across different domains. This loss plays a critical role in ensuring that the content latent space remains domain-invariant, allowing the classifier to generalize effectively to target domains. Given an input batch ${ℬ}_{x}={\left\{x_{i}\right\}}_{i=1}^{\left|{ℬ}_{x}\right|}$and its counterpart ${ℬ}_{x}^{\prime }={\left\{x_{i}^{\prime }\right\}}_{i=1}^{\left|{ℬ}_{x}^{\prime }\right|}$, where $\left|{ℬ}_{x}|=|{ℬ}_{x}^{\prime }\right|$, the invariance loss ${ℒ}_{inv}\left(\theta ,{ℬ}_{x},{ℬ}_{x}^{\prime }\right)$ is defined based on the discrepancy between the predictions of these two feature batches (Equation 16):


$$\begin{array}{l} {ℒ}_{inv}\left(\theta ,{ℬ}_{x},{ℬ}_{x}^{\prime }\right)=\frac{1}{\left|{ℬ}_{x}\right|}\overset{\left|{ℬ}_{x}\right|}{\underset{i=1}{\sum }}d\left[f\left(x_{i},\theta \right),f\left(x_{i}^{\prime },\theta \right)\right] \end{array}$$ (16)

where $x_{i}\in {ℬ}_{x}$, $x_{j}\in {ℬ}_{x}^{\prime }$, and *d*(·, ·) is the distance metric over predictions.

#### 4.4.3 Fairness loss

The fairness loss ${ℒ}_{fair}\left(\theta ,ℬ\right)$ ensures equitable performance across sensitive subgroups defined by the sensitive attribute, where *g* defined in Definition 1 measures the deviation from demographic parity for sample *x*_*i*_. It seeks to mitigate biases in the model's predictions by enforcing statistical parity between predictions for different sensitive groups (Equation 17).


$$\begin{array}{ll} {ℒ}_{fair}\left(\theta ,ℬ\right) & =|\frac{1}{\left|ℬ\right|}\underset{\left(x_{i},z_{i}\right)\in ℬ}{\sum }g\left(x_{i},z_{i},\theta \right)| \end{array}$$ (17)

In FFDGUA, a batch of data ℬ is first sampled and used to generate an augmented batch ${ℬ}_{aug}$ through the transformation model trained during Stage 1. These batches are subsequently utilized to compute the three loss functions: classification loss, invariance loss, and fairness loss. The model parameters ***θ*** are updated based on these losses using the Adam optimizer. The contributions of the invariance and fairness losses are balanced through the hyperparameters λ_1_ and λ_2_, respectively, which are updated following the parameter optimization.

**Figure 6:**
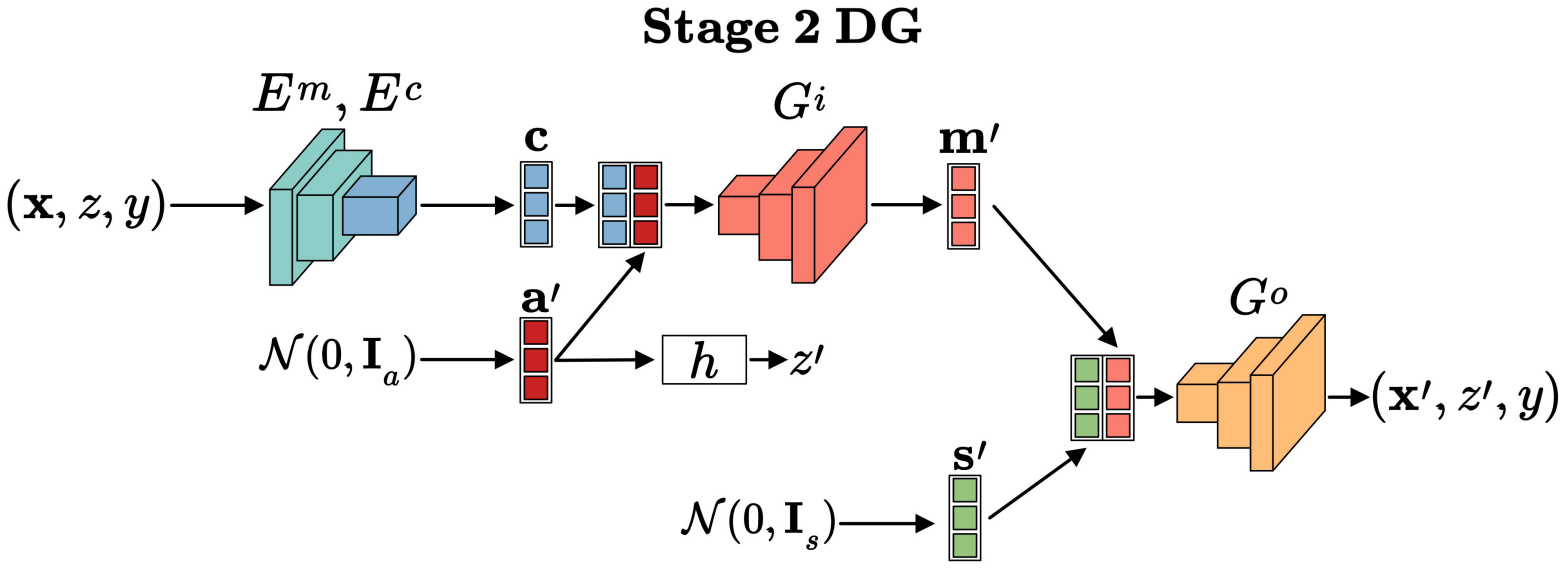
For DG, data are generated through an underlying domain transformation model based on invariant content factors encoded from stage 1 and randomly sampled style and sensitive factors that encode synthetic domains.

**Figure 7:**
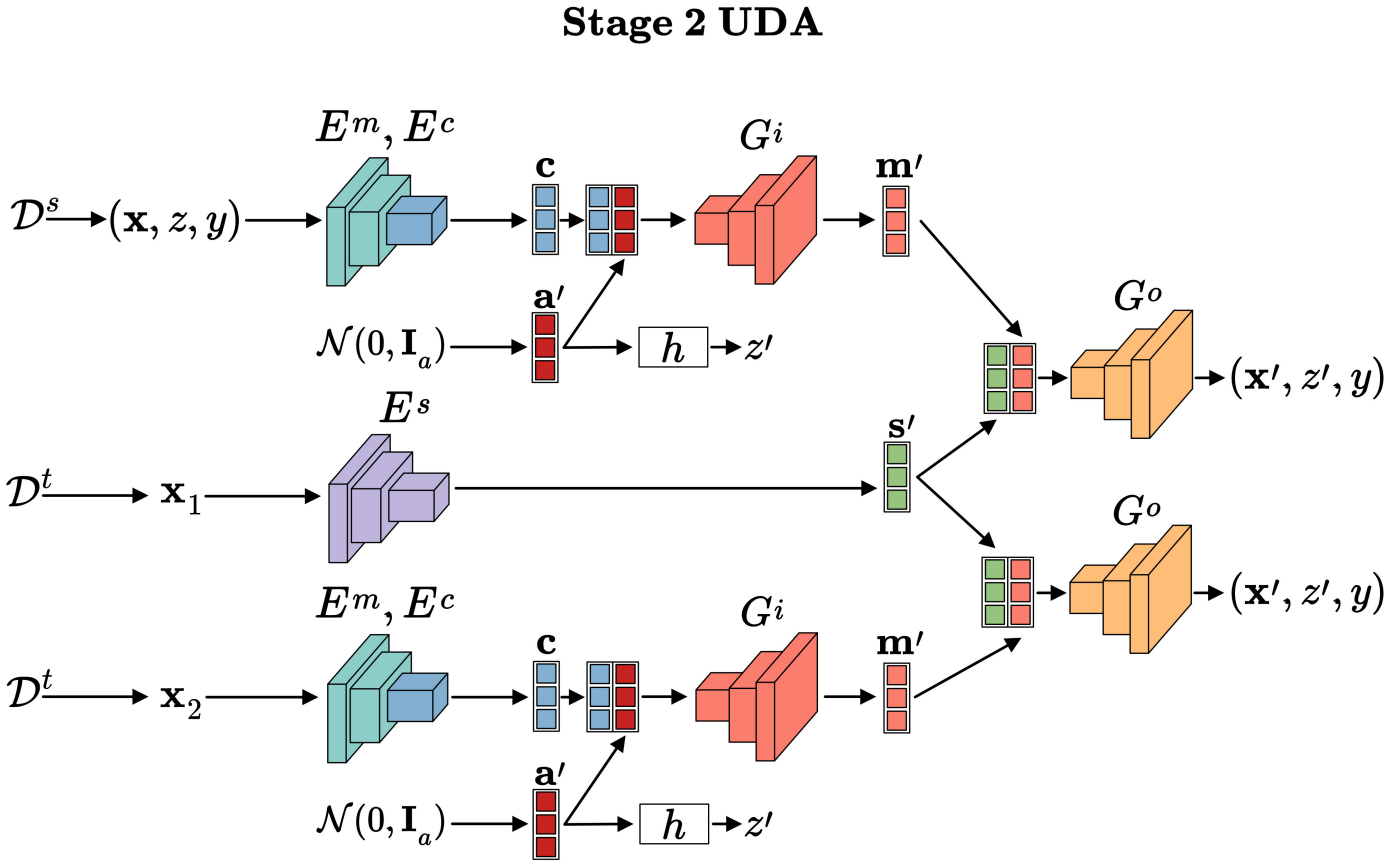
For UDA, it is similar to DG, but style factors are encoded from unlabeled target domain samples.

As illustrated in Figures 6, 7, the proposed framework is designed to address both domain generalization and unsupervised domain adaptation. The pseudo-codes for these two scenarios are provided in Algorithms 1, 2, detailing the specific procedures for each task.

Algorithm 1. **Fairness-aware Classification for Domain Generalization**

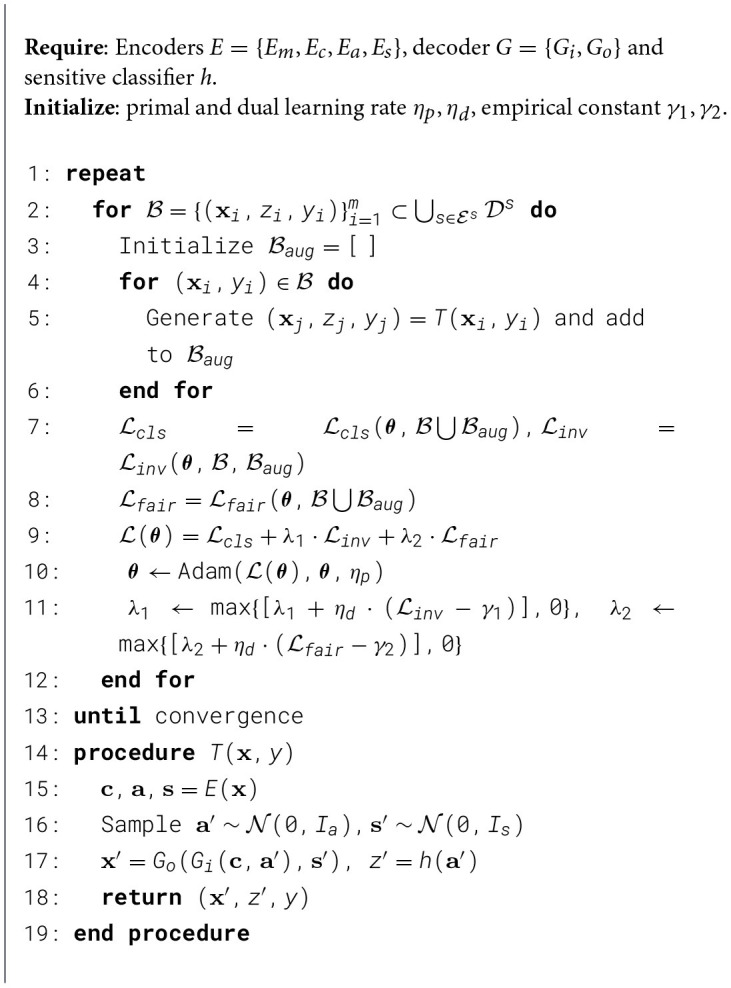

Algorithm 2. **Fairness-aware classification for unsupervised domain adaptation.**

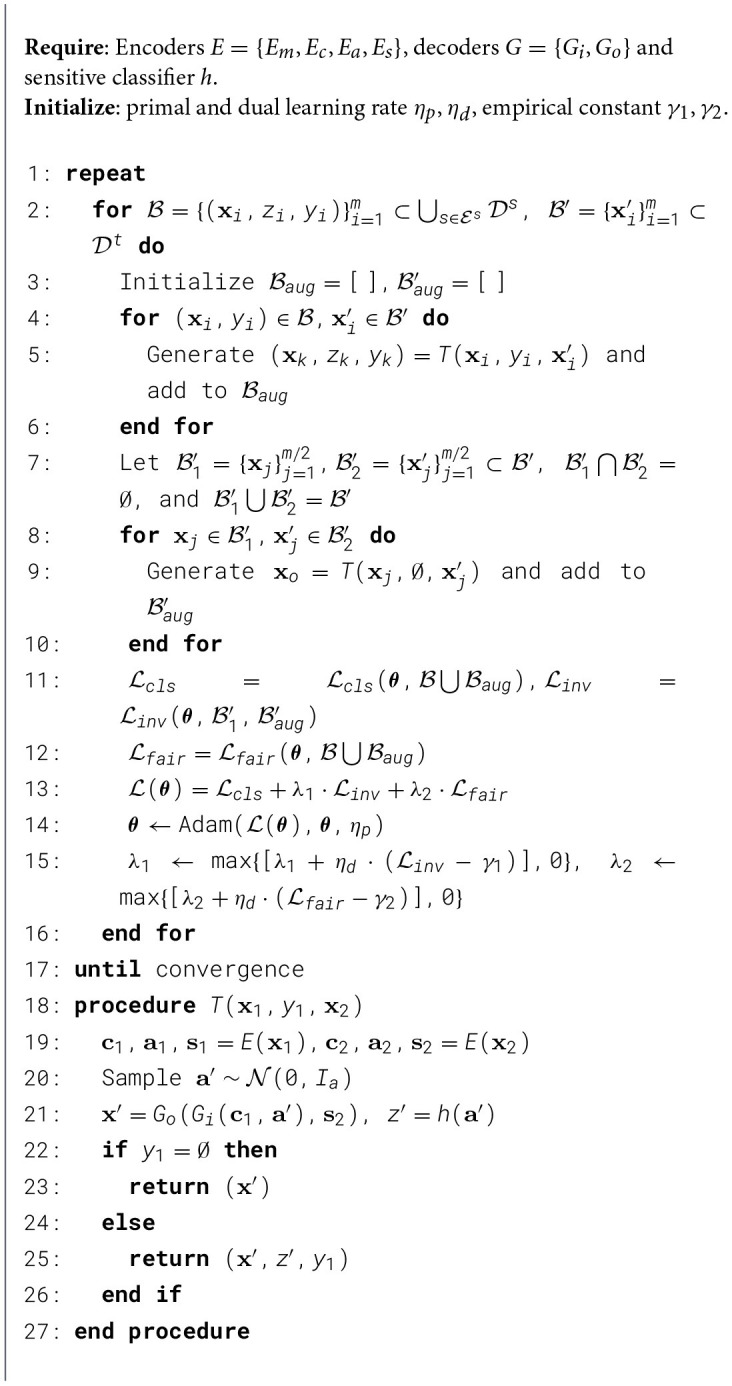

#### 4.4.4 Implementation for domain generalization

Given finite source domains under domain generalization, to enhance generalization to unseen target domains, we train the invariant classifier using synthetic domains generated through transformation model *T*. These synthetic domains are generated by introducing two key randomization operations: (1) random style factors $s^{\prime }\sim N\left(0,I_{s}\right)$ and (2) random sensitive factors $a^{\prime }\sim N\left(0,I_{a}\right)$, as depicted in Figure 6. The synthetic sensitive attribute *z*′is then derived via *z*′ = *h*(***a*′**), while preserving the content factor **c** encoded from the original sample (**x**, *z, y*). Through decoders *G*_*i*_, *G*_*o*_, these components produce augmented samples (***x*′**, *z*′, *y*) that retain the original class labels. Under Assumption 2 and Definition 3, this synthesis process ensures invariance in both accuracy and fairness between original and synthetic domains, as formalized by the constraints in Equations 12, 13 - requiring consistent classifier behavior across domain transformations while breaking spurious attribute-feature correlations.

As shown in Algorithm 1, data batches are sampled and utilized to generate augmented batches in lines 2-6. These augmented batches are subsequently used to compute the three loss components: classification, invariance, and fairness losses (lines 7–8). Lines 10–11 involve updating the parameters θ using the Adam optimizer, with the hyperparameters λ_1_ and λ_2_ controlling the contributions of the invariance and fairness losses, respectively. The hyperparameters are updated in line 11. Finally, the transformation models defined in lines 14–19 are applied during the generation of augmented batches, ensuring diversity and robustness in the training data.

#### 4.4.5 Implementation for unsupervised domain adaptation

Under unsupervised domain adaptation, to enhance generalization to target domains with unlabeled samples, we train the invariant classifier using samples generated via the transformation model *T*. Unlike DG, the style factors **s**′ are encoded from unlabeled target domain samples, while sensitive factors $a^{\prime }\sim N\left(0,I_{a}\right)$ are randomly sampled. The sensitive attribute *z*′is computed as *z*′ = *h*(***a*′**), and the content factor **c** is encoded from source or target samples. Decoders *G*_*i*_ and *G*_*o*_ then generate augmented samples (***x*′**, *z*′, *y*). When **c** is from the target domain, labels *y* are unknown but not required, as these samples contribute only to the invariance loss, which does not require explicit class labels.

As illustrated in Algorithm 2, data batches are sampled and processed through domain transformation operations in lines 2–10 to create augmented batches. These synthetic samples are subsequently used to compute three key objective terms: the classification loss ${ℒ}_{cls}$, domain invariance loss ${ℒ}_{inv}$, and fairness constraint loss ${ℒ}_{fair}$ (line 11–13). The model parameters ***θ*** are optimized using the Adam optimizer with learning rates η_*p*_ and η_*d*_, where the hyperparameters λ_1_ and λ_2_ control the relative importance of the invariance and fairness objectives respectively (line 14). Adaptive adjustment of these hyperparameters occurs through dual variable updates based on constraints (line 15). Here, the transformation model *T* (lines 18-27) generating augmented batches by disentangling and recombining latent factors, which aligns with Figure 7.

#### 4.4.6 Time complexity

To analyze the time complexity, let *m* be the batch size, *n* be the number of training iterations, and $\left|{ℰ}^{s}\right|$ be the number of source domains.

In Algorithm 1, for each batch (line 2), the algorithm iterates over *m* samples to generate augmented data (lines 4–6), which involves encoding (*E*_*m*_, *E*_*c*_, *E*_*a*_, *E*_*s*_), decoding (*G*_*i*_, *G*_*o*_), and predicting sensitive attributes (*h*). Each such transformation takes *O*(1) with fixed-size networks, assuming that the input data dimension is constant across all processed batches, so the cost per batch is *O*(*m*). The loss computations (line 7) also scale linearly with *m*. Parameter updates (lines 8–10) involve standard optimizer steps, also costing *O*(*m*). Therefore, each iteration has a time complexity of *O*(*m*), and the total time complexity across *n* iterations is *O*(*mn*). The complexity is independent of the number of domains $\left|{ℰ}^{s}\right|$, as each batch samples uniformly from all source domains.

In Algorithm 2, in each iteration (lines 2–16), the algorithm samples *m* labeled data points from the source domains and *m* unlabeled samples from the target domain. For each pair (*x*_*i*_, *x*_*j*_) (lines 4-6), augmented data is generated using the transformation model, which includes encoding (*E*_*m*_, *E*_*c*_, *E*_*a*_, *E*_*s*_), decoding (*G*_*i*_, *G*_*o*_), and sensitive attribute prediction (*h*), each takes *O*(1) time due to fixed-size networks and a constant input data dimension. Additionally, extra augmentations are performed using pairs from the target domain (lines 8-10), introducing another *O*(*m*) operations. Losses (classification, invariance, fairness) are computed over *O*(*m*) samples (line 11-12), and optimization steps are *O*(*m*) as well. Therefore, each iteration has a time complexity of *O*(*m*), leading to a total time complexity of *O*(*mn*).

## 5 Experiments

To comprehensively evaluate the effectiveness of FFDGUA, we conducted experiments across multiple benchmarks covering diverse domain. These benchmarks were strategically chosen to cover a wide range of domain characteristics and sensitive attributes, ensuring a thorough evaluation of the framework's performance.

### 5.1 Empirical settings

#### 5.1.1 Datasets

To assess the performance of FFDGUA, we perform experiments on four diverse datasets: ccMNIST, FairFace, YFCC100M-FDG, and New York Stop-and-Frisk (NYSF). Of these, NYSFis a tabular dataset, whereas ccMNIST, FairFace, and YFCC100M-FDG are image datasets. (a) ccMNIST. The ccMNISTdataset is constructed from the original MNIST dataset (LeCun et al., 1998) by incorporating color attributes into the digits and their backgrounds. It consists of grayscale images of handwritten digits (0–9) that are categorized into two binary classes: digits 0–4 are assigned to class 0, while digits 5–9 are assigned to class 1, as described in the ColoredMNIST methodology (Arjovsky et al., 2019). To enable domain generalization research, the dataset is divided into three distinct domains based on digit color (red, green, and blue). Each domain exhibits a unique correlation between the class label and the sensitive attribute (background color), with correlation coefficients of 0.9, 0.7, and 0.0 for the red, green, and blue domains, respectively. These specific values were selected to simulate varying degrees of dependence shifts. Because our experimental setup uses a leave-one-domain-out protocol where each domain alternately serves as the target domain while the other two act as source domains, this combination of values evaluates the model under diverse and challenging shift scenarios. For instance, testing on the 0.0 correlation domain evaluates whether the model has successfully disentangled the sensitive attribute from the class label after training on highly biased sources (0.9 and 0.7), while testing on the 0.9 or 0.7 domains evaluates the framework's ability to maintain fairness and accuracy when generalizing to a highly biased target domain from sources with differing or absent biases. The dataset contains a total of 70,000 images. (b) FairFace. The FairFace dataset (Karkkainen and Joo, 2021) comprises 108,501 facial images and is curated to ensure balanced representation across seven racial groups: Black (B), East Asian (E), Indian (I), Latino (L), Middle Eastern (M), Southeast Asian (S), and White (W). For our experiments, each racial group is treated as an individual domain. The binary classification label is based on age (whether ≥50 years or < 50 years), with gender serving as the sensitive attribute. (c) YFCC100M-FDG. The YFCC100M-FDGdataset is a curated subset of the YFCC100M dataset (Thomee et al., 2016), developed by Yahoo Labs. It contains 90,000 images, randomly sampled and categorized into three domains based on the year of capture: before 1999 (*d*_0_), between 2000 and 2009 (*d*_1_), and from 2010 to 2014 (*d*_2_). Each domain includes 30,000 images. The binary class label indicates whether an image is classified as indoor or outdoor. The sensitive attribute is determined using latitude and longitude, distinguishing images captured in North America from those taken elsewhere. (d) NYSF. The NYSF dataset (Koh et al., 2021) is a real-world dataset comprising records of police stops in New York City during 2011. The objective is to predict whether a pedestrian suspected of carrying a weapon was indeed found in possession of one. This dataset highlights societal biases, particularly against African Americans. The data is divided into five geographic domains corresponding to sub-city regions: Manhattan (M), Brooklyn (B), Queens (Q), Bronx (R), and Staten Island (S). Race, classified as Black or non-Black, is considered the sensitive attribute.

#### 5.1.2 Evaluation metrics

We assess the framework's performance using three metrics, Accuracy (*Acc*) and two metrics aimed at quantifying fairness: Demographic Parity (*DP*) (Dwork et al., 2011) and the Area Under the ROC Curve (*AUC*_*fair*_) for sensitive subgroups (Ling et al., 2003). Notably, *AUC*_*fair*_ differs from the conventional *AUC* utilized in classification, which relies on TPR and FPR. Instead, *AUC*_*fair*_ is grounded in the nonparametric *Mann-Whitney U* test, where fairness is defined as the probability of a classifier assigning a higher score to a randomly selected sample **x**_−1_ from one sensitive subgroup than to a sample **x**_1_ from another subgroup being equal to the reverse probability (Zhao and Chen, 2019; Calders et al., 2013). A *DP* value approaching 1 indicates higher fairness, whereas an *AUC*_*fair*_ value of 0.5 suggests unbiased predictions.

#### 5.1.3 Architectures

We have two sets of network architectures. One is designed for ccMNIST, FairFace, and YFCC100M-FDG, and the other is tailored for the NYSFdataset.

For the ccMNIST, FairFace, and YFCC100M-FDGdatasets, all images are resized to 224 × 224. *E*_*m*_ and *E*_*c*_ share the same structure, each consisting of four convolutional layers. The first layer has 64 filters, while the remaining three have 128 filters. The kernel sizes are (7, 7), (4, 4), (3, 3), (3, 3) for layers 1 to 4, respectively. The stride for the second layer is (2, 2), and for all other layers, it is (1, 1). The first three layers use ReLU as the activation function, and the last convolutional layer has no activation function. *E*_*s*_ and *E*_*a*_ also share the same structure, consisting of six convolutional layers and an adaptive average pooling layer with output size 1 positioned between the last two convolutional layers. The filter numbers are 64, 128, 256, 256, 256, and 2, respectively. The kernel sizes are (7, 7), (4, 4), (4, 4), (4, 4), (4, 4), (1, 1), and the strides are (1, 1), (2, 2), (2, 2), (2, 2), (2, 2), (1, 1). ReLU is used as the activation function for the first five layers, while the last layer has no activation function. *G*_*o*_ and *G*_*i*_ have nearly identical structures, differing only in their output sizes, which are 3 for *G*_*o*_ and 128 for *G*_*i*_. Both consist of two parts. The first part comprises four convolutional layers, with an upsampling layer (scale factor 2.0) between the second and third layers. The numbers of filters are 128, 128, 64, and 3, with kernel sizes (3, 3), (3, 3), (5, 5), (7, 7). The strides for all layers are (1, 1). The activation functions for the first and third layers are ReLU, the fourth layer uses Tanh, and the second layer has no activation function. The second part contains three fully connected layers with 256, 256, and 512 neurons. ReLU is the activation function for the first two layers, while the last layer has no activation function. *D*_*o*_ consists of four convolutional layers followed by an average pooling layer with a kernel size of 3, stride of 2, and padding of [1, 1]. The convolutional layers have 64, 128, 256, and 1 filters, with kernel sizes (4, 4) for the first three layers and (1, 1) for the last layer. The strides are (2, 2) for the first three layers and (1, 1) for the last layer. The first three layers use LeakyReLU, while the remaining layers have no activation function. *D*_*i*_ comprises a single fully connected layer with an input size of 112 and an output size of 64, using ReLU as the activation function. *h* is a single fully connected layer with an input size of 2 and an output size of 1, with a Sigmoid activation function. *f* consists of two parts. The first is a ResNet-50 model (He et al., 2016), and the second is a fully connected layer with an input size of 2048 and an output size of 2.

For the NYSFdataset, *E*_*m*_ consists of two fully connected layers with 32 neurons and an output size of 16. The first layer uses ReLU, while the second layer has no activation function. *E*_*s*_ also comprises two fully connected layers with 32 neurons and an output size of 2. The first layer uses ReLU, and the second layer has no activation function. *G*_*o*_ consists of two fully connected layers with 32 neurons and an output size of 51. The first layer uses ReLU, while the second layer has no activation function. *D*_*o*_ is composed of two fully connected layers with 32 neurons and an output size of 16. The first layer uses ReLU, and the second layer has no activation function. *E*_*c*_ comprises two fully connected layers with 16 neurons and an output size of 8. The first layer uses ReLU, and the second layer has no activation function. *E*_*a*_ is made of two fully connected layers with 8 neurons and an output size of 2. The first layer uses ReLU, and the second layer has no activation function. *G*_*i*_ includes two fully connected layers with 16 neurons and an output size of 16. The first layer uses ReLU, and the second layer has no activation function. *D*_*i*_ consists of two fully connected layers with 8 neurons and an output size of 8. The first layer uses ReLU, and the second layer has no activation function. *h* is a single fully connected layer with an input size of 2 and an output size of 1, using a Sigmoid activation function. *f* has two components. The first is made of three fully connected layers with 32 neurons and an output size of 32. The first two layers use ReLU, while the output layer has no activation function. The second component is a fully connected layer with an input size of 32 and an output size of 32, without an activation function.

#### 5.1.4 Model selection

To evaluate the performance of our approach, we adopt the leave-one-domain-out validation protocol, which is suitable for both domain generalization and unsupervised domain adaptation. This methodology, as supported by Robey et al. (2021) and identified as one of the three selection methods by Gulrajani and Lopez-Paz (2020), systematically withholds one domain from the training process. The reported performance is averaged across all withheld domains, providing a comprehensive evaluation of the framework's domain generalization and unsupervised domain adaptation capabilities.

#### 5.1.5 Effectiveness of *T*

**Figure 8:**
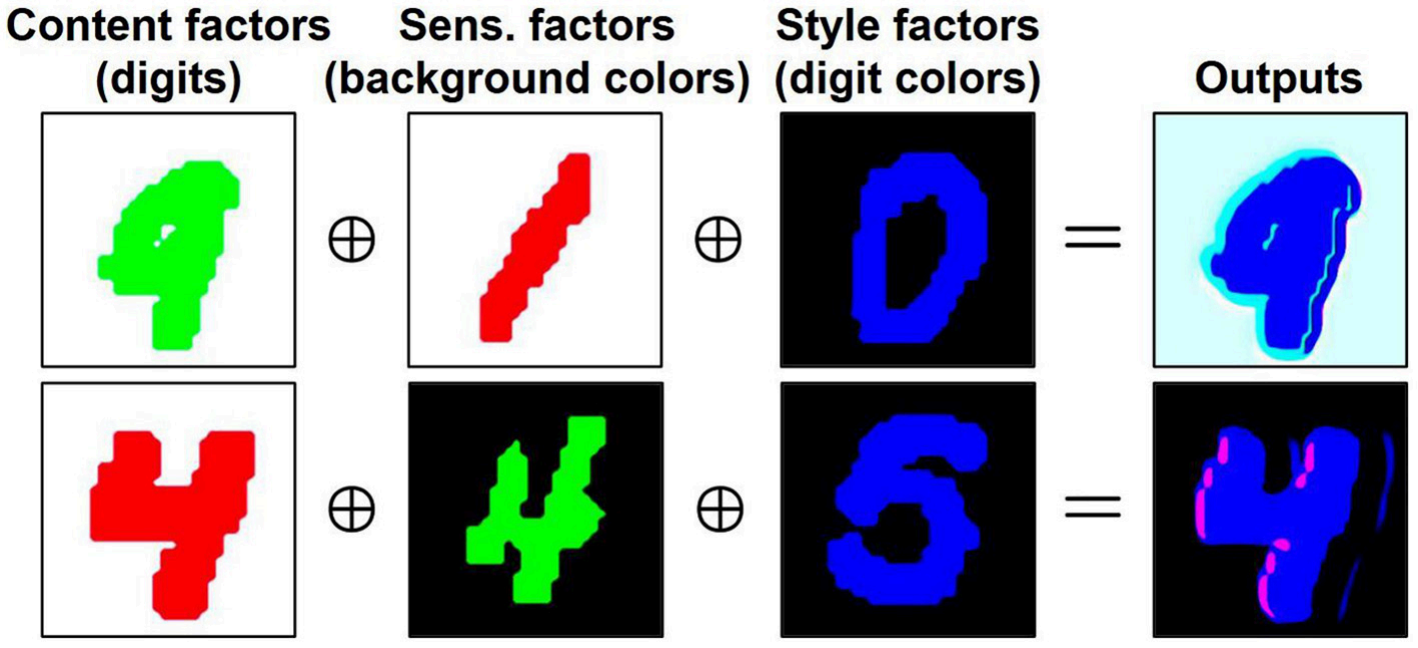
Examples of results obtained by generating images using latent factors encoded from three different images.

To further evaluate the effectiveness of *T*, we draw inspiration from Huang et al. (2018) and train a separate transformation model for each domain. We then generate output images by combining distinct latent factors from different domains. Using ccMNISTas an illustrative example, we independently train three transformation models ${\left\{T^{i}\right\}}_{i=1}^{3}$, each specific to a different domain. Each transformation model *T*^*i*^ comprises unique encoders: $E_{c}^{i}$ for content, $E_{a}^{i}$ for sensitive, and $E_{s}^{i}$ for style. As shown in Figure 8, an output image is synthesized by the generator *G* using a content factor (digit class, extracted as $E_{c}^{1}\left(x^{1}\right)$), a sensitive factor (background color, extracted as $E_{a}^{2}\left(x^{2}\right)$), and a style factor (digit color, extracted as $E_{s}^{3}\left(x^{3}\right)$) from images belonging to different domains. As a result, the generated image retains the digit structure of **x**^1^, the background color of **x**^2^, and the digit color of **x**^3^, with controlled variations.

#### 5.1.6 Hyperparameter search

We follow the hyperparameter setup from MUNIT (Huang et al., 2018). Specifically, the learning rate is set to 0.0001, the number of iterations is 600, 000, and the batch size is 1. The loss weights for training *T* are selected from {1, 5, 10}. The optimal values are determined to be β_1_ = 10, β_2_ = 1, β_3_ = 1, and β_4_ = 1. These values are chosen by monitoring the validation set loss and selecting the β configuration with the lowest loss. For training the classifier *f*, hyperparameters are selected as follows. The learning rate is chosen from {0.000005, 0.00001, 0.00005, 0.0001, 0.0005}. The parameter η is chosen from {0.01, 0.05, 0.1}, while γ is chosen from {0.01, 0.025, 0.05}. The parameter λ is selected from {0.1, 1, 10, 20}, and the batch size is chosen from {22, 64, 80, 128, 512, 1024, 2048}. The number of iterations is selected from {500, 1000, …, 8000}for the ccMNIST and NYSFdatasets, and from {300, 600, …, 7, 800, 8, 000} for the FairFaceand YFCC100M-FDGdatasets. The optimal hyperparameters are as follows: the learning rate is 0.00005, η_1_ = η_2_ = 0.05, γ_1_ = γ_2_ = 0.025, and λ_1_ = λ_2_ = 1. The batch size is 64 for the ccMNISTand YFCC100M-FDGdatasets, 22 for the FairFacedataset, and 1024 for the NYSFdataset. The number of iterations for each dataset and domain is as follows: For the ccMNIST dataset: 3, 000, 500, and 7, 000 for domains R, G, and B, respectively. For the FairFace dataset: 7, 200, 7, 200, 7, 800, 8, 000, 6, 600, 7, 200, and 6, 900 for domains B, E, I, L, M, S, and W, respectively. For the YFCC100M-FDGdataset: 7, 200, 6, 000, and 6, 900 for domains *d*_0_, *d*_1_, and *d*_2_, respectively. For the NYSFdataset: 500, 3, 500, 4, 000, 1, 500, and 8, 000 for domains R, B, M, Q, and S, respectively. The accuracy and fairness metrics are monitored on the validation set, and the hyperparameter configurations yielding the best results are selected. The grid search space for all baseline methods is identical to that used for our proposed method.

FFDGUA was comprehensively evaluated against 19 state-of-the-art baselines, providing a thorough analysis of its performance in comparison to existing methods. Details of experiments for domain generalization and unsupervised domain adaptation are provided in Sections 5.2, 5.3 respectively.

### 5.2 Domain generalization experiments

#### 5.2.1 Baselines

In our evaluation, FFDGUA is compared against 19 baseline methods for domain generalization, which are grouped into two categories based on their primary focus and methodological approach: (a) twelve state-of-the-art *domain generalization* methods: CORAL (Sun and Saenko, 2016), MMD (Li H. et al., 2018), DANN (Ganin et al., 2016), CDANN (Li Y. et al., 2018), DDG (Zhang et al., 2022), MBDG (Robey et al., 2021),ColorJitter, ERM (Vapnik, 1999), IRM (Arjovsky et al., 2019), GroupDRO (Sagawa et al., 2020), Mixup (Yan et al., 2020), and MLDG (Li D. et al., 2018) where ColorJitter is a simple function in *PyTorch* that applies random adjustments to the brightness, contrast, saturation, and hue of images; (b) seven *fairness-aware domain generalization* methods: EIIL (Creager et al., 2021), FarconVAE (Oh et al., 2022), FCR (An et al., 2022), FTCS (Roh et al., 2023), FATDM (Pham et al., 2023), DDG-FC, and MBDG-FC where DDG-FC and MBDG-FC are adaptations of DDG (Zhang et al., 2022) and MBDG (Robey et al., 2021) with additional fairness constraints in Equation 17 integrated into their classification frameworks.

#### 5.2.2 Domain samples availability

In domain generalization, we take one domain as the target domain and all the other domains as the source domains for each dataset. During training, 80% labeled samples from each source domain are used, and target domain samples are entirely excluded from training. The transformation model is trained on all source domain data, while the classifier is trained on $|{ℰ}^{s}|-1$ source domains and validated on the held-out domain.

#### 5.2.3 Data augmentation

**Figure 9:**
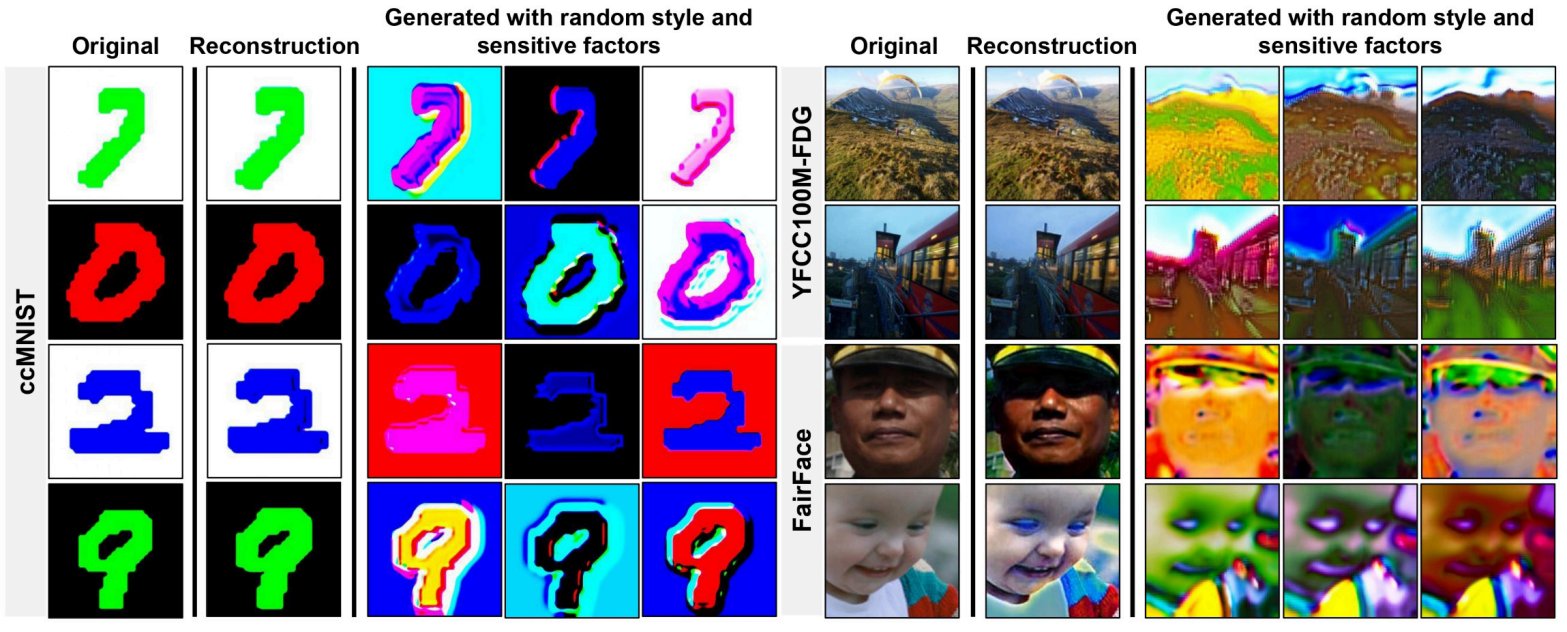
Visualizations of images undergoing reconstruction and the transformation model *T* with randomly sampled style and sensitive factors. Source images taken from FairFace Dataset Karkkainen and Joo (2021).

We present a visualization of the augmented samples with random variations in Figure 9. The first column (Original) displays images sampled directly from the datasets. The second column (Reconstruction) contains images generated from latent factors encoded from the original samples. These reconstructed images closely resemble their corresponding original images, preserving their fundamental semantic information. The last three columns showcase images generated by using the content factors encoded from the original samples while randomly sampling the style and sensitive factors from their respective Gaussian distributions. Although the generated images retain the core content factors of the original samples, their style and sensitive factors undergo substantial modifications. These synthetic domain samples contribute to improving the classifier's generalization ability (*f*) across unseen source domains. This visualization highlights the effectiveness of the transformation model *T* in extracting latent factors and generating diverse, realistic transformations of data across domains.

#### 5.2.4 Quantitative results

Comprehensive experiments reveal that FFDGUA consistently outperforms baseline methods by a notable margin. Across all four tables, each column represents the performance on a specific target domain, with the remaining domains serving as source domains. The number following the domain name indicates the difference in demographic parity for that domain. The rightmost column reports the average performance across all domains. As illustrated in Table 2, for the FairFacedataset, FFDGUA achieves the highest average performance in terms of both accuracy and fairness metrics across all domains. Specifically, the proposed method improves fairness by 3% in *DP* and 2% in *AUC*_*fair*_, while achieving a marginally higher accuracy (an improvement of 0.19%) compared to the best baseline for individual metrics. Similarly, as shown in Table 3, for the YFCC100M-FDGdataset, FFDGUA demonstrates substantial improvements in fairness metrics, with an 8% increase in *DP* and a 4% increase in *AUC*_*fair*_, while maintaining a comparable accuracy, improving by 0.35% over the best baseline. For the ccMNISTand NYSFdatasets, presented in Tables 4, 5, FFDGUA achieves the best average fairness performance. On ccMNIST, it shows a 5% improvement in *DP* and a 1% improvement in *AUC*_*fair*_. On NYSF, it improves *DP* by 4% and *AUC*_*fair*_ by 2%. While the average accuracy on these datasets is not the highest, it remains very close to the best baselines, with a difference of only 1.05% on ccMNISTand 0.87% on NYSF.

**Table 2:** Domain generalization performance on FairFace.

Methods	*DP* ↑/*AUC*_*fair*_ ↓/*Acc* ↑			
	(B, 0.91)	(E, 0.87)	(I, 0.58)	(W, 0.49)
ColorJitter	0.64/0.64/93.47	0.41/0.68/95.62	0.44/0.63/92.99	0.34/0.64/92.07
ERM; Vapnik (1999)	0.67/0.58/91.89	0.43/0.64/95.69	0.50/0.59/93.28	0.39/0.61/92.82
IRM; Arjovsky et al. (2019)	0.63/0.58/93.39	0.32/0.63/95.12	0.45/0.59/92.01	0.32/0.66/90.54
GDRO; Sagawa et al. (2020)	0.71/0.57/89.81	0.46/0.61/95.26	0.50/0.59/93.27	0.48/0.60/92.50
Mixup; Yan et al. (2020)	0.58/0.59/92.46	0.40/0.61/93.31	0.42/0.59/**93.42**	0.43/0.61/92.98
MLDG; Li D. et al. (2018)	0.63/0.58/92.71	0.41/0.62/95.59	0.51/0.60/93.35	0.47/0.59/92.82
CORAL; Sun and Saenko (2016)	0.69/0.58/92.09	0.34/0.64/**95.91**	0.53/0.59/93.35	0.50/0.60/92.47
MMD; Li H. et al. (2018)	0.69/0.56/93.87	0.45/**0.57**/94.68	0.27/**0.57**/89.88	0.39/0.68/91.75
DANN; Ganin et al. (2016)	0.46/0.61/91.80	0.53/0.85/91.54	0.38/0.63/90.09	0.11/0.66/86.80
CDANN; Li Y. et al. (2018)	0.62/0.59/91.22	0.43/0.66/94.75	0.43/0.61/92.41	0.35/0.67/90.19
DDG; Zhang et al. (2022)	0.60/0.59/91.76	0.36/0.63/95.52	0.49/0.59/92.35	0.51/0.60/91.34
MBDG; Robey et al. (2021)	0.60/0.58/91.29	0.46/0.63/95.01	0.52/0.58/92.77	0.30/0.62/91.05
DDG-FC; Zhang et al. (2022)	0.61/0.58/92.27	0.39/0.64/95.51	0.45/0.58/93.38	0.48/0.62/92.45
MBDG-FC; Robey et al. (2021)	0.70/0.56/92.12	0.35/0.60/95.54	**0.56/0.57**/92.41	0.32/0.60/91.50
EIIL; Creager et al. (2021)	0.88/0.59/84.75	0.69/0.71/92.86	0.47/**0.57**/86.93	0.46/0.65/86.53
FarconVAE; Oh et al. (2022)	0.93/**0.54**/89.61	0.72/0.63/91.50	0.42/0.58/87.42	0.51/0.60/86.40
FCR; An et al. (2022)	0.81/0.59/79.66	0.60/0.69/89.22	0.40/0.62/79.15	0.39/0.63/82.33
FTCS; Roh et al. (2023)	0.75/0.60/80.00	0.66/0.65/88.11	0.49/0.65/82.15	0.40/0.60/79.66
FATDM; Pham et al. (2023)	0.93/0.57/92.20	0.80/0.65/92.89	0.52/0.60/92.22	0.46/0.63/92.56
FFDGUA	**0.94**/0.55/**93.91**	**0.87**/0.60/**95.91**	0.48/**0.57**/92.55	**0.52/0.58/93.02**
**Methods**	*DP* ↑/*AUC*_*fair*_ ↓/*Acc* ↑			
	**(L, 0.48)**	**(M, 0.87)**	**(S, 0.39)**	**Avg**
ColorJitter	0.39/0.70/91.77	0.36/0.65/92.79	0.35/0.69/91.89	0.42/0.66/92.94
ERM; Vapnik (1999)	0.57/0.62/91.96	0.34/0.62/92.51	0.68/0.59/93.48	0.51/0.61/93.08
IRM; Arjovsky et al. (2019)	0.41/0.63/92.06	0.34/0.65/92.47	0.55/0.59/91.81	0.43/0.62/92.48
GDRO; Sagawa et al. (2020)	0.54/0.62/91.59	0.45/0.63/91.75	0.72/0.59/93.65	0.55/0.60/92.55
Mixup; Yan et al. (2020)	0.55/0.61/93.43	0.31/0.62/**93.52**	0.91/0.58/93.20	0.51/0.60/93.19
MLDG; Li D. et al. (2018)	0.53/0.62/92.99	0.35/0.62/92.45	0.71/0.57/**93.85**	0.51/0.60/93.39
CORAL; Sun and Saenko (2016)	0.56/**0.59**/92.62	0.43/0.63/92.23	0.74/0.58/93.77	0.54/0.60/93.21
MMD; Li H. et al. (2018)	0.55/0.61/92.53	0.48/0.62/91.07	0.66/0.59/92.58	0.50/0.60/92.34
DANN; Ganin et al. (2016)	0.39/0.67/90.82	**0.65**/0.88/91.46	0.80/0.57/88.20	0.47/0.70/90.10
CDANN; Li Y. et al. (2018)	0.42/0.61/92.42	0.27/0.67/91.07	0.52/0.82/88.32	0.43/0.66/91.48
DDG; Zhang et al. (2022)	0.44/0.62/93.46	0.37/0.64/91.36	0.63/0.58/93.40	0.49/0.61/92.74
MBDG; Robey et al. (2021)	0.56/0.61/93.49	0.38/0.64/92.23	0.67/0.56/93.12	0.50/0.60/92.71
DDG-FC; Zhang et al. (2022)	0.50/0.62/92.42	0.42/0.95/92.70	0.76/0.59/**93.85**	0.52/0.61/93.23
MBDG-FC; Robey et al. (2021)	0.57/0.62/91.89	0.49/0.63/90.67	0.74/0.57/93.24	0.53/0.60/92.48
EIIL; Creager et al. (2021)	0.49/**0.59**/88.39	0.52/0.63/84.96	**0.98/0.55**/89.99	0.64/0.61/87.78
FarconVAE; Oh et al. (2022)	**0.58**/0.60/88.70	0.54/**0.58**/85.62	0.92/0.56/90.00	0.66/**0.58**/88.46
FCR; An et al. (2022)	0.38/0.66/85.22	0.51/0.66/82.16	0.72/0.60/88.01	0.54/0.63/83.68
FTCS; Roh et al. (2023)	0.42/0.65/79.64	0.49/0.68/81.15	0.75/0.62/75.69	0.57/0.64/80.91
FATDM; Pham et al. (2023)	0.51/0.63/93.33	0.55/0.65/92.23	0.92/0.57/92.36	0.67/0.61/92.54
FFDGUA	**0.58/0.59/93.73**	0.54/0.62/92.61	**0.98/0.55**/92.26	**0.70/0.58/93.42**

Bold is the best; underline is the second best.

**Table 3:** Domain generalization performance on YFCC100M-FDG.

Methods	*DP* ↑/*AUC*_*fair*_ ↓/*Acc* ↑			
	(*d*_0_, 0.73)	(*d*_1_, 0.84)	(*d*_2_, 0.72)	Avg
ColorJitter	0.67/0.57/57.47	0.67/0.61/82.43	0.65/0.64/87.88	0.66/0.61/75.93
ERM; Vapnik (1999)	0.81/0.58/40.51	0.71/0.66/83.91	0.89/0.59/82.06	0.80/0.61/68.83
IRM; Arjovsky et al. (2019)	0.76/0.58/50.51	0.87/0.60/73.26	0.70/0.57/82.78	0.78/0.58/68.85
GDRO; Sagawa et al. (2020)	0.80/0.59/53.43	0.73/0.60/87.56	0.79/0.65/83.10	0.78/0.62/74.70
Mixup; Yan et al. (2020)	0.82/0.57/61.15	0.79/0.63/78.63	0.89/0.60/85.18	0.84/0.60/74.99
MLDG; Li D. et al. (2018)	0.75/0.67/49.56	0.71/0.57/89.45	0.71/0.57/87.51	0.72/0.60/75.51
CORAL; Sun and Saenko (2016)	0.80/0.58/58.96	0.72/0.64/91.66	0.70/0.64/89.28	0.74/0.62/79.97
MMD; Li H. et al. (2018)	0.79/0.59/61.51	0.71/0.64/91.15	0.79/0.60/86.69	0.76/0.61/79.87
DANN; Ganin et al. (2016)	0.70/0.78/47.71	0.79/0.53/84.80	0.77/0.59/58.50	0.75/0.64/63.67
CDANN; Li Y. et al. (2018)	0.74/0.58/55.87	0.70/0.65/87.06	0.72/0.63/85.76	0.72/0.62/76.23
DDG; Zhang et al. (2022)	0.81/0.57/60.08	0.74/0.66/92.53	0.71/0.59/**95.02**	0.75/0.61/82.54
MBDG; Robey et al. (2021)	0.79/0.58/60.46	0.73/0.67/94.36	0.71/0.59/93.48	0.74/0.61/82.77
DDG-FC; Zhang et al. (2022)	0.76/0.58/59.96	0.83/0.58/**96.80**	0.82/0.59/86.38	0.80/0.58/81.04
MBDG-FC; Robey et al. (2021)	0.80/0.58/62.31	0.72/0.63/94.73	0.80/**0.53**/87.78	0.77/0.58/81.61
EIIL; Creager et al. (2021)	**0.87**/0.55/56.74	0.76/0.54/68.99	0.87/0.78/72.19	0.83/0.62/65.98
FarconVAE; Oh et al. (2022)	0.67/0.61/51.21	0.90/0.59/72.40	0.85/0.55/74.20	0.81/0.58/65.93
FCR; An et al. (2022)	0.62/0.70/55.32	0.63/0.66/70.89	0.66/0.78/70.58	0.64/0.71/65.60
FTCS; Roh et al. (2023)	0.72/0.60/60.21	0.79/0.59/79.96	0.69/0.60/72.99	0.73/0.60/71.05
FATDM; Pham et al. (2023)	0.80/0.55/61.56	0.88/0.56/90.00	0.86/0.60/89.12	0.84/0.57/80.22
FFDGUA	**0.87/0.53/62.56**	**0.94/0.52**/93.36	**0.93/0.53**/93.43	**0.92/0.53/83.12**

Bold is the best; underline is the second best.

**Table 4:** Domain generalization performance on ccMNIST.

Methods	*DP* ↑/*AUC*_*fair*_ ↓/*Acc* ↑			
	(R, 0.11)	(G, 0.43)	(B, 0.87)	Avg
ColorJitter	0.11/0.95/90.59	0.44/0.71/87.62	0.87/0.66/86.33	0.47/0.77/88.18
ERM; Vapnik (1999)	0.12/0.91/98.00	0.43/0.78/98.07	0.89/0.64/95.64	0.48/0.78/97.24
IRM; Arjovsky et al. (2019)	0.21/0.97/75.50	0.28/0.64/92.74	0.76/0.63/80.05	0.42/0.75/82.76
GDRO; Sagawa et al. (2020)	0.12/0.92/98.19	0.43/0.75/98.17	0.90/0.65/95.03	0.48/0.77/97.13
Mixup; Yan et al. (2020)	0.12/0.92/97.89	0.41/0.79/98.00	0.93/0.65/96.09	0.49/0.79/97.32
MLDG; Li D. et al. (2018)	0.11/0.91/98.52	0.43/0.77/**98.67**	0.87/0.62/93.76	0.46/0.77/96.98
CORAL; Sun and Saenko (2016)	0.11/0.91/**98.69**	0.42/0.79/98.30	0.87/0.64/93.74	0.47/0.78/96.91
MMD; Li H. et al. (2018)	0.11/0.92/**98.69**	0.41/0.73/97.72	0.93/0.59/95.37	0.48/0.75/97.26
DANN; Ganin et al. (2016)	0.14/0.87/85.94	0.17/0.90/84.93	0.76/0.63/84.04	0.36/0.80/84.97
CDANN; Li Y. et al. (2018)	0.19/0.90/93.03	0.60/0.89/71.92	0.77/0.63/84.03	0.52/0.81/82.99
DDG; Zhang et al. (2022)	0.11/0.91/98.26	0.42/0.77/98.14	0.96/0.60/**97.02**	0.50/0.76/**97.81**
MBDG; Robey et al. (2021)	0.12/0.93/98.47	0.42/0.81/97.62	0.90/0.64/96.01	0.48/0.79/97.37
DDG-FC; Zhang et al. (2022)	0.11/0.91/96.69	0.42/0.75/96.09	**0.97**/0.58/95.66	0.50/0.75/96.14
MBDG-FC; Robey et al. (2021)	0.13/0.91/98.07	0.45/0.76/96.09	0.94/0.64/95.42	0.50/0.77/96.52
EIIL; Creager et al. (2021)	0.15/0.94/81.00	0.26/0.98/82.67	0.62/0.98/71.68	0.34/0.97/78.45
FarconVAE; Oh et al. (2022)	0.11/0.94/94.40	0.43/0.77/82.61	**0.97**/0.59/76.22	0.50/0.77/84.41
FCR; An et al. (2022)	0.12/0.90/79.00	0.56/0.75/77.65	0.82/0.67/70.35	0.50/0.77/75.67
FTCS; Roh et al. (2023)	0.10/0.93/83.16	0.52/0.69/78.65	0.80/0.66/72.51	0.66/0.69/95.59
FATDM; Pham et al. (2023)	0.17/0.86/96.00	0.92/0.64/95.55	0.90/**0.57**/95.23	0.66/0.67/95.59
FFDGUA	**0.23/0.84**/96.15	**0.98/0.58**/97.94	0.92/**0.57**/96.19	**0.71/0.66**/96.76

Bold is the best; underline is the second best.

**Table 5:** Domain generalization performance on NYSF.

Methods	*DP* ↑/*AUC*_*fair*_ ↓/*Acc* ↑		
	(R, 0.93)	(B, 0.85)	(M, 0.81)
ERM; Vapnik (1999)	0.91/0.53/60.21	0.90/0.54/58.93	0.92/0.54/59.49
IRM; Arjovsky et al. (2019)	0.98/0.52/61.61	0.94/**0.52**/56.89	0.92/0.53/59.64
GDRO; Sagawa et al. (2020)	0.81/0.56/58.73	0.89/0.55/59.44	0.87/0.55/**62.57**
Mixup; Yan et al. (2020)	0.96/0.53/**62.63**	0.90/0.54/58.96	0.92/0.54/58.29
MLDG; Li D. et al. (2018)	0.96/0.52/61.81	0.90/0.55/58.11	0.93/0.53/58.27
CORAL; Sun and Saenko (2016)	0.95/0.52/62.17	0.93/0.54/58.06	0.95/0.53/58.84
MMD; Li H. et al. (2018)	0.91/0.53/60.34	0.89/0.55/58.47	0.92/0.54/59.31
DANN; Ganin et al. (2016)	0.83/0.52/40.80	**0.96**/0.55/54.55	0.88/**0.52**/59.19
CDANN; Li Y. et al. (2018)	0.95/0.52/57.61	0.94/0.54/56.97	0.87/**0.52**/59.59
DDG; Zhang et al. (2022)	0.92/0.52/56.52	0.92/0.54/58.21	0.92/0.53/60.91
MBDG; Robey et al. (2021)	0.96/0.52/55.96	0.90/0.70/51.52	**0.96**/0.53/58.74
DDG-FC; Zhang et al. (2022)	0.95/0.52/54.53	0.93/0.53/59.32	0.92/0.52/60.08
MBDG-FC; Robey et al. (2021)	0.96/0.55/55.93	0.91/0.54/55.50	0.90/0.53/57.37
EIIL; Creager et al. (2021)	0.95/0.52/58.28	0.92/0.54/56.76	0.83/0.54/59.47
FarconVAE; Oh et al. (2022)	0.90/0.53/60.52	0.89/0.55/**60.30**	0.82/0.56/60.31
FCR; An et al. (2022)	0.91/0.55/56.21	0.88/0.56/55.12	0.77/0.55/58.15
FTCS; Roh et al. (2023)	0.92/0.54/58.23	0.84/0.56/49.23	0.80/0.56/55.48
FATDM; Pham et al. (2023)	0.93/0.52/59.32	0.86/0.58/59.01	0.85/0.53/60.45
FFDGUA	**0.99/0.50**/62.01	**0.96/0.52**/58.37	0.92/**0.52**/59.49
**Methods**	*DP* ↑/*AUC*_*fair*_ ↓/*Acc* ↑		
	**(Q, 0.59)**	**(S, 0.62)**	**Avg**
ERM; Vapnik (1999)	0.88/0.57/62.48	0.86/0.61/54.54	0.90/0.56/59.13
IRM; Arjovsky et al. (2019)	0.87/0.54/55.81	0.89/0.54/57.00	0.92/0.53/58.19
GDRO; Sagawa et al. (2020)	0.86/0.57/62.92	0.77/0.64/60.44	0.84/0.57/**60.82**
Mixup; Yan et al. (2020)	0.93/0.53/61.34	0.84/0.61/53.07	0.91/0.55/58.86
MLDG; Li D. et al. (2018)	0.89/0.56/62.85	0.85/0.59/54.42	0.91/0.55/59.10
CORAL; Sun and Saenko (2016)	0.95/0.53/61.45	0.88/0.54/52.08	0.93/0.53/58.52
MMD; Li H. et al. (2018)	0.88/0.56/62.48	0.81/0.61/57.73	0.88/0.56/59.67
DANN; Ganin et al. (2016)	0.96/0.53/63.60	0.86/0.56/58.96	0.90/0.54/55.42
CDANN; Li Y. et al. (2018)	0.97/0.54/**64.25**	0.74/0.60/57.73	0.89/0.54/59.23
DDG; Zhang et al. (2022)	0.89/0.55/56.68	0.84/0.58/54.91	0.90/0.54/57.44
MBDG; Robey et al. (2021)	0.96/0.52/60.73	0.90/**0.52**/52.45	0.93/0.56/55.88
DDG-FC; Zhang et al. (2022)	0.92/0.54/59.90	0.90/0.57/57.45	0.92/0.53/58.26
MBDG-FC; Robey et al. (2021)	0.94/0.52/61.04	0.91/0.53/52.57	0.92/0.53/56.48
EIIL; Creager et al. (2021)	0.84/0.55/52.18	0.95/0.59/55.74	0.90/0.54/56.49
FarconVAE; Oh et al. (2022)	0.97/0.56/61.30	0.86/0.58/60.70	0.89/0.56/60.62
FCR; An et al. (2022)	0.85/0.59/59.15	0.82/0.55/51.45	0.83/0.56/56.02
FTCS; Roh et al. (2023)	0.89/0.58/57.15	0.84/0.56/56.15	0.86/0.56/55.25
FATDM; Pham et al. (2023)	0.85/0.52/60.35	0.88/**0.52**/59.22	0.87/0.53/59.67
FFDGUA	**0.99/0.50**/59.11	**0.98**/0.53/**60.77**	**0.97/0.51**/59.95

Bold is the best; underline is the second best.

#### 5.2.5 Ablation studies

**Figure 10:**
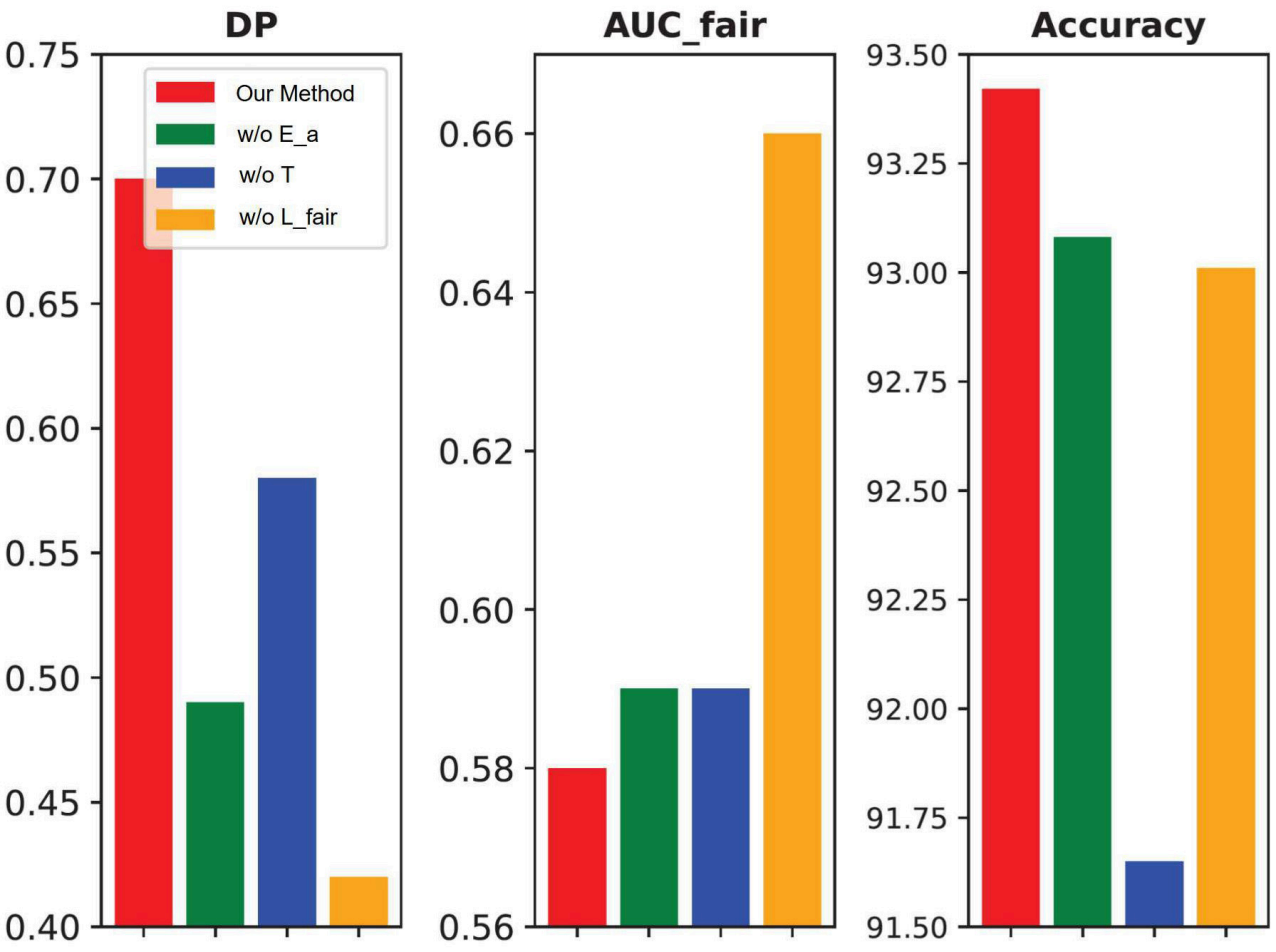
Domain generalization ablation study on FairFace, with averaged results plotted across all domains.

We conduct three ablation studies to evaluate the robustness of FFDGUA on the FairFacedataset. (1) In FFDGUA w/o *E_a_*, we modify the encoder within *T* by restricting its output to only latent content and style factors, omitting the sensitive factor. (2) FFDGUA w/o *T* bypasses data augmentation in synthetic domains via *T*, relying solely on the classifier *f* constrained by fairness notions as defined in Definition 1. (3) In FFDGUA w/o ℒfair, the fairness constraint on *f* is removed, and the fairness loss term ${ℒ}_{fair}$ is eliminated from the optimization objective. The performance of these ablation studies is presented in Figure 10. More results are in Tables 6–9. The results demonstrate that disentangling data into three distinct latent factors, content, style, and sensitive factors, combined with a model structure that explicitly accounts for these factors, enhances generalization performance under covariate and dependence shifts. Furthermore, generating data in synthetic domains with randomized fairness dependence patterns proves to be an effective strategy for achieving fairness invariance across domains.

**Table 6:** Domain generalization ablation studies results on ccMNIST.

Methods	*DP* ↑/*AUC*_*fair*_ ↓/*Acc* ↑			
	(R, 0.11)	(G, 0.43)	(B, 0.87)	Avg
FFDGUA w/o *E_a_*	0.23/0.98/94.89	0.11/0.92/98.19	0.42/0.72/95.28	0.25/0.87/96.12
FFDGUA w/o *T*	0.21/0.92/96.74	0.15/0.86/96.95	0.48/0.57/96.05	0.28/0.79/96.58
FFDGUA w/o ℒfair	0.22/0.91/96.63	0.44/0.75/97.90	0.97/0.61/96.01	0.54/0.76/96.85

**Table 7:** Domain generalization ablation studies results on FairFace.

Methods	*DP* ↑/*AUC*_*fair*_ ↓/*Acc* ↑			
	(B, 0.91)	(E, 0.87)	(I, 0.58)	(M, 0.87)
FFDGUA w/o *E_a_*	0.68/0.57/93.07	0.43/0.60/95.55	0.37/0.59/92.26	0.49/0.62/92.61
FFDGUA w/o *T*	0.83/0.56/92.81	0.50/0.56/95.12	0.42/0.59/92.34	0.39/0.68/91.46
FFDGUA w/o ℒfair	0.59/0.58/92.92	0.36/0.62/95.55	0.42/0.62/93.35	0.38/0.72/92.27
**Methods**	*DP* ↑/*AUC*_*fair*_ ↓/*Acc* ↑			
	**(S, 0.39)**	**(W, 0.49)**	**(L, 0.48)**	**Avg**
FFDGUA w/o *E_a_*	0.69/0.56/93.28	0.35/0.58/92.18	0.47/0.63/92.62	0.49/0.59/93.08
FFDGUA w/o *T*	0.92/0.56/87.87	0.52/0.59/90.78	0.53/0.59/91.19	0.58/0.59/91.65
FFDGUA w/o ℒfair	0.42/0.67/92.17	0.34/0.72/91.88	0.40/0.70/92.96	0.42/0.66/93.01

**Table 8:** Domain generalization ablation studies results on YFCC100M-FDG.

Methods	*DP* ↑/*AUC*_*fair*_ ↓/*Acc* ↑			
	(*d*_0_, 0.73)	(*d*_1_, 0.84)	(*d*_2_, 0.72)	Avg
FFDGUA w/o *E_a_*	0.69/0.57/43.09	0.83/0.63/89.68	0.89/0.54/87.70	0.80/0.58/73.49
FFDGUA w/o *T*	0.82/0.56/47.21	0.83/0.63/73.10	0.82/0.53/72.95	0.82/0.57/64.42
FFDGUA w/o ℒfair	0.72/0.69/54.24	0.92/0.64/94.35	0.92/0.64/93.20	0.86/0.66/80.59

**Table 9:** Domain generalization ablation studies results on NYSF.

Methods	*DP* ↑/*AUC*_*fair*_ ↓/*Acc* ↑		
	(R, 0.93)	(B, 0.85)	(M, 0.81)
FFDGUA w/o *E_a_*	0.95/0.52/55.78	0.97/0.51/55.30	0.95/0.53/58.29
FFDGUA w/o *T*	0.95/0.52/61.36	0.91/0.54/57.67	0.89/0.55/60.68
FFDGUA w/o ℒfair	0.95/0.52/63.72	0.87/0.55/58.86	0.89/0.54/60.61
**Methods**	*DP* ↑/*AUC*_*fair*_ ↓/*Acc* ↑		
	**(Q, 0.59)**	**(S, 0.62)**	**Avg**
FFDGUA w/o *E_a_*	0.92/0.54/57.61	0.90/0.59/52.82	0.94/0.53/55.96
FFDGUA w/o *T*	0.97/0.52/59.33	0.87/0.57/55.40	0.92/0.54/58.89
FFDGUA w/o ℒfair	0.83/0.57/64.17	0.89/0.58/56.51	0.89/0.55/60.77

#### 5.2.6 Fairness-accuracy tradeoff

**Figure 11:**
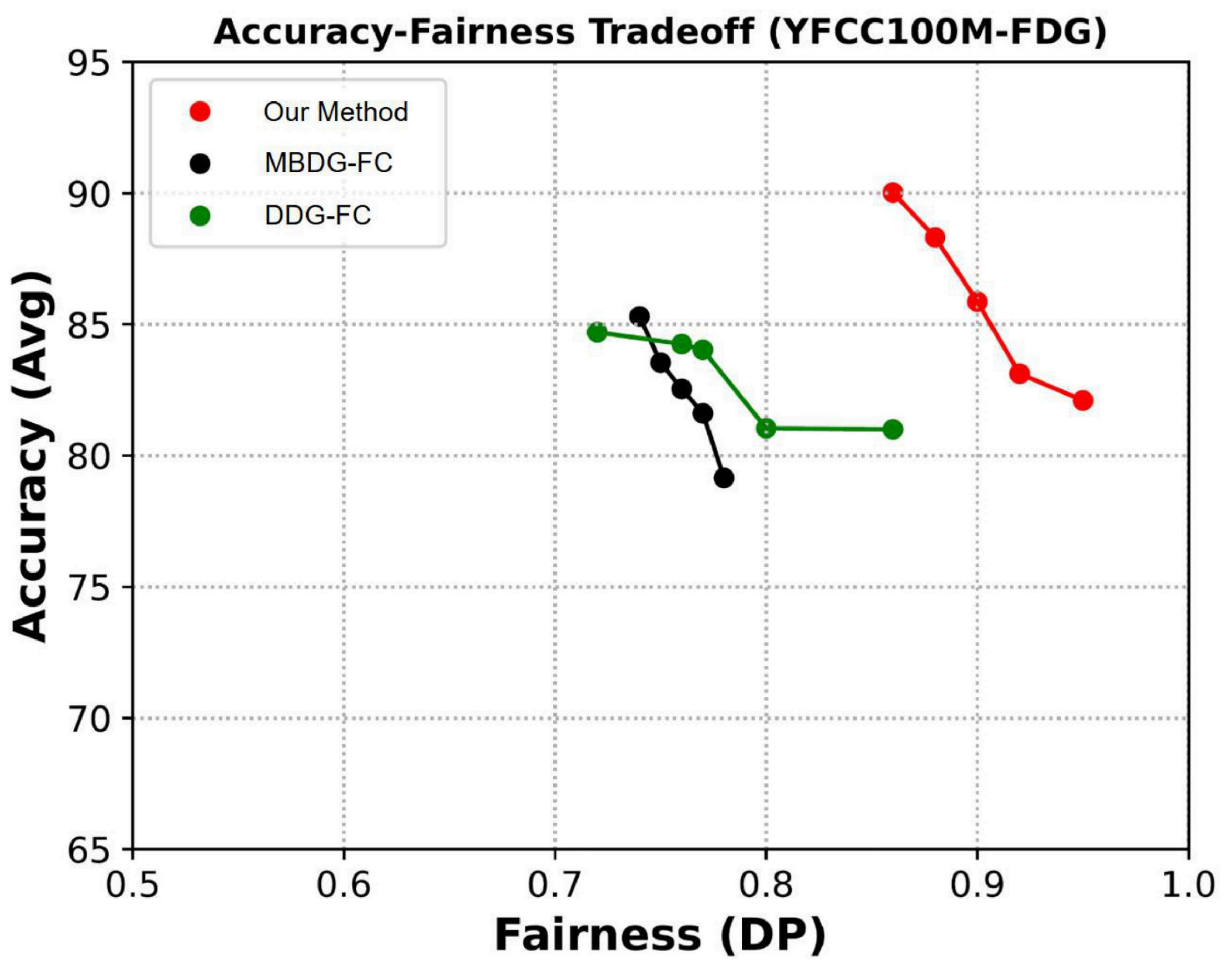
Accuracy-fairness tradeoff results on FairFace(left) and YFCC100M-FDG(right), evaluated over a range of λ_2_ values.

In Algorithm 1, the parameter λ_2_ (lines 8 and 10) serves as the regularization coefficient for the fairness loss. To analyze its impact, we conduct additional experiments, varying λ_2_ over the range λ_2_∈{0.01, 0.05, 0.1, 1, 10}, and examine the resulting tradeoff between accuracy and fairness. Our findings indicate that increasing λ_2_ enhances model fairness both within individual domains and on average, but comes at the cost of reduced accuracy. Conversely, smaller values of λ_2_ lead to higher accuracy but compromise fairness. Evaluations on FairFaceand YFCC100M-FDGare presented in Figure 11. Results positioned on the top-right of the figure correspond to optimal tradeoffs, representing a balance between accuracy and fairness. The plotted results reflect the average performance across all target domains.

### 5.3 Unsupervised domain adaptation experiments

#### 5.3.1 Baselines

In our evaluation, FFDGUA is compared against 8 baseline methods for unsupervised domain adaptation, which are grouped into two categories based on their primary focus and methodological approach: (a) six state-of-the-art *domain generalization* methods which can be easily adapted to unsupervised domain adaptation since they can utilize the unlabeled sample from the target domain: CORAL (Sun and Saenko, 2016), MMD (Li H. et al., 2018), DANN (Ganin et al., 2016), CDANN (Li Y. et al., 2018), DDG (Zhang et al., 2022), and MBDG (Robey et al., 2021); (b) three unsupervised domain adaptation methods: DAD (Peng et al., 2024), TD-BLS (Zhang et al., 2025), and DLRE (Zhao et al., 2025); (c) two *fairness-aware domain generalization* methods: DDG-FC and MBDG-FC which are adaptations of DDG (Zhang et al., 2022) and MBDG (Robey et al., 2021) with additional fairness constraints in Equation 17 integrated into their classification frameworks.

#### 5.3.2 Domain samples availability

In unsupervised domain adaptation, same as in domain generalization, we take one domain as the target domain and all the other domains as the source domains for each dataset. During training, 80% labeled samples in source domains are available. 50% unlabeled samples in the target domain are also available. In other words, features of 50% samples in the target domain are available, while their ground truth labels and sensitive attributes are unavailable during training. For training the transformation model, we use all the available samples in source domains and target domains. For training the classifier, as we are using leave-one-domain-out validation strategy, the parameters are trained on $|{ℰ}^{s}|-1$ source domains and the target domain, which are $\left|{ℰ}^{s}\right|$ domains in total.

#### 5.3.3 Data augmentation

**Figure 12:**
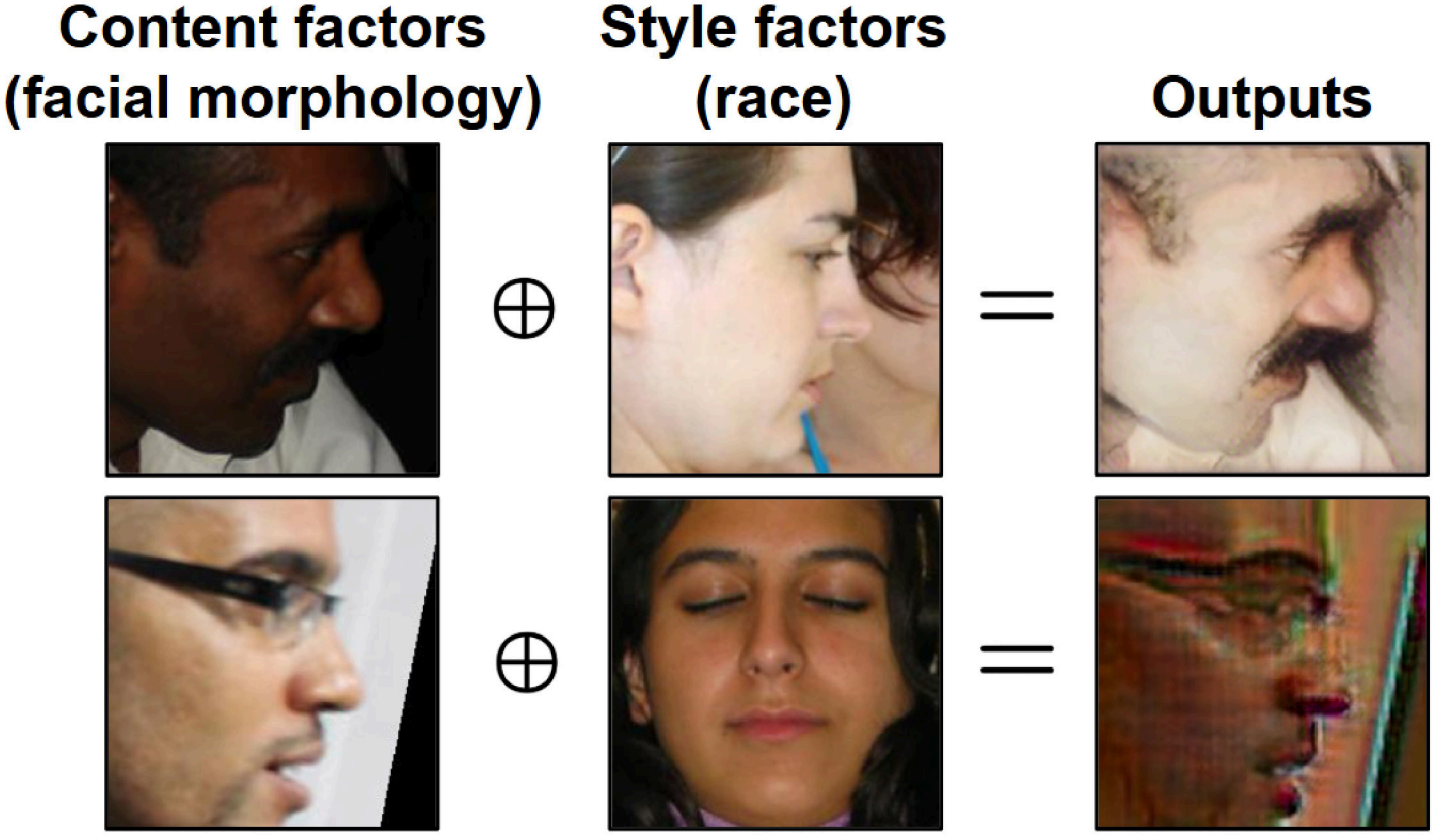
Visualizations of images generated by the transformation model *T*, where content and style factors are encoded from different images, and sensitive factors are randomly sampled. Source images taken from FairFace Dataset Karkkainen and Joo (2021).

We provide a visualization of augmented samples with random variations in Figure 12. The first two columns (Original) show images directly sampled from the datasets. The third column displays images generated by combining content factors encoded from the first column, style factors encoded from the second column, and sensitive factors randomly sampled from a Gaussian distribution. While these generated images preserve the core content factor of the original samples, their style and sensitive factors undergo significant modifications. These generated samples enhance the classifier's generalization ability (*f*) within the domain from which the style factors were encoded. This approach strengthens generalization performance in target domains, even when only a limited number of unlabeled samples are available during training. The visualization underscores the effectiveness of the transformation model *T* in extracting latent factors and producing diverse, realistic transformations across domains.

#### 5.3.4 Quantitative result

Comprehensive experimental results demonstrate that FFDGUA consistently outperforms baseline methods by a significant margin. Similar to the domain generalization tables, each column in the result tables corresponds to the performance on a specific target domain, with the remaining domains serving as source domains. The demographic parity difference for each domain is indicated after the domain name, while the final column provides the average performance across all domains. As shown in Table 10, for YFCC100M-FDG, FFDGUA achieves the best performance across all evaluation metrics. Specifically, it improves *DP* by 1%, accuracy by 0.62%, and achieves parity in *AUC*_*fair*_ with the best baseline methods. In the NYSFdataset, illustrated in Table 11, FFDGUA achieves the highest average accuracy and fairness metrics across all domains. Notably, it enhances *DP* by 1%, *AUC*_*fair*_ by 1%, and accuracy by 2.42%. For the ccMNISTdataset, as shown in Table 12, FFDGUA achieves the best average accuracy, outperforming the best baseline by 0.36%. Additionally, it matches the best baseline methods in *DP* and achieves the second-best *AUC*_*fair*_, which is only 1% lower than the top-performing baseline. In Table 13, for the FairFacedataset, FFDGUA delivers the best accuracy, exceeding the best baseline by 0.26%, and achieves parity with the best baseline methods in *AUC*_*fair*_. Although the average *DP* of FFDGUA ranks third, the two methods with better *DP* demonstrate accuracy levels that are over 20% lower than FFDGUA.

**Table 10:** Unsupervised domain adaptation performance on YFCC100M-FDG.

Methods	*DP* ↑/*AUC*_*fair*_ ↓/*Acc* ↑			
	(*d*_0_, 0.73)	(*d*_1_, 0.84)	(*d*_2_, 0.72)	Avg
CORAL; Sun and Saenko (2016)	0.82/0.51/82.68	0.93/**0.51**/88.19	0.92/0.52/93.73	0.89/**0.51**/88.20
MMD; Li H. et al. (2018)	0.85/0.51/76.66	0.93/**0.51**/89.37	0.92/0.51/89.39	0.90/**0.51**/85.14
DANN; Ganin et al. (2016)	0.79/0.76/76.64	0.89/0.75/40.81	0.90/0.88/72.33	0.86/0.80/63.26
CDANN; Li Y. et al. (2018)	0.79/0.75/49.18	0.85/0.80/62.95	0.49/0.78/70.35	0.71/0.77/60.83
DDG; Zhang et al. (2022)	0.80/0.52/71.73	0.93/0.52/93.55	0.92/0.52/92.59	0.89/0.52/85.96
MBDG; Robey et al. (2021)	0.83/0.51/76.36	0.93/**0.51**/95.50	**0.93**/0.51/94.82	0.90/**0.51**/88.89
DAD; Peng et al. (2024)	0.78/0.52/79.79	0.90/0.53/87.22	0.92/0.52/86.90	0.87/0.52/84.63
TD-BLS; Zhang et al. (2025)	0.84/0.53/82.61	0.91/0.53/85.55	0.90/0.54/85.68	0.88/0.54/84.61
DLRE; Zhao et al. (2025)	0.87/0.51/82.28	0.92/0.52/85.73	0.91/0.51/86.03	0.90/0.52/88.01
DDG-FC; Zhang et al. (2022)	0.80/0.51/65.48	**0.94/0.51**/95.05	0.92/**0.50**/94.35	0.89/**0.51**/84.96
MBDG-FC; Robey et al. (2021)	**0.91**/0.51/**83.44**	0.90/**0.51**/85.84	0.89/0.51/80.05	0.90/**0.51**/83.11
FFDGUA	0.85/**0.50**/77.23	0.93/**0.51/95.55**	**0.93**/0.51/**95.73**	**0.91/0.51/89.51**

Bold is the best; underline is the second best.

**Table 11:** Unsupervised domain adaptation performance on NYSF.

Methods	*DP* ↑/*AUC*_*fair*_ ↓/*Acc* ↑		
	(R, 0.93)	(B, 0.85)	(M, 0.81)
CORAL; Sun and Saenko (2016)	0.97/0.52/61.96	0.93/0.51/55.77	0.95/0.51/57.87
MMD; Li H. et al. (2018)	0.88/0.52/59.46	0.89/0.51/56.91	0.91/0.51/55.65
DANN; Ganin et al. (2016)	0.98/0.52/56.04	0.89/0.52/57.27	0.92/0.51/55.30
CDANN; Li Y. et al. (2018)	0.95/0.51/60.16	0.90/0.52/56.75	0.93/0.51/55.10
DDG; Zhang et al. (2022)	0.98/0.51/62.45	0.92/0.51/56.10	**0.98**/0.51/60.21
MBDG; Robey et al. (2021)	0.93/0.51/63.07	0.87/**0.50**/58.02	0.94/0.51/59.40
DAD; Peng et al. (2024)	0.95/0.51/64.65	0.93/**0.50**/54.28	0.94/0.52/53.69
TD-BLS; Zhang et al. (2025)	0.94/0.51/64.43	0.92/0.52/54.84	0.92/0.51/55.39
DLRE; Zhao et al. (2025)	0.97/0.51/62.53	**0.95**/0.51/55.23	0.95/0.53/56.16
DDG-FC; Zhang et al. (2022)	0.98/0.51/52.44	**0.95**/0.51/55.30	0.95/0.51/60.13
MBDG-FC; Robey et al. (2021)	0.95/0.51/62.62	0.90/**0.50**/58.09	0.95/0.51/59.74
FFDGUA	**1.00/0.50/65.18**	0.93/**0.50/59.89**	0.96/**0.50/60.70**
**Methods**	*DP* ↑/*AUC*_*fair*_ ↓/*Acc* ↑		
	**(Q, 0.59)**	**(S, 0.62)**	**Avg**
CORAL; Sun and Saenko (2016)	0.97/0.51/59.27	0.92/0.51/55.28	0.95/0.52/58.03
MMD; Li H. et al. (2018)	0.82/0.51/59.49	0.87/0.52/54.59	0.88/0.52/57.22
DANN; Ganin et al. (2016)	0.82/0.51/61.26	0.90/0.52/56.07	0.90/0.52/57.19
CDANN; Li Y. et al. (2018)	0.77/0.52/60.19	0.94/0.52/56.31	0.90/0.52/57.70
DDG; Zhang et al. (2022)	0.92/0.51/61.19	0.88/0.52/55.38	0.94/0.51/59.07
MBDG; Robey et al. (2021)	0.94/**0.50**/61.68	**0.99**/0.52/52.68	0.93/0.51/58.97
DAD; Peng et al. (2024)	0.95/0.51/60.12	0.95/0.53/52.21	0.94/0.51/56.99
TD-BLS; Zhang et al. (2025)	0.95/**0.50**/60.69	0.93/0.54/54.67	0.93/0.52/58.00
DLRE; Zhao et al. (2025)	0.97/0.51/61.22	0.96/0.53/56.14	0.96/0.52/58.26
DDG-FC; Zhang et al. (2022)	0.87/0.51/57.47	0.96/0.51/55.68	0.94/0.51/56.20
MBDG-FC; Robey et al. (2021)	0.94/**0.50**/61.79	0.96/0.52/51.99	0.94/0.51/58.85
FFDGUA	**0.99/0.50/62.03**	0.97/**0.50/59.66**	**0.97/0.50/61.49**

Bold is the best; underline is the second best.

**Table 12:** Unsupervised domain adaptation performance on ccMNIST.

Methods	*DP* ↑/*AUC*_*fair*_ ↓/ *Acc* ↑			
	(R, 0.11)	(G, 0.43)	(B, 0.87)	Avg
CORAL; Sun and Saenko (2016)	0.13/0.55/96.84	0.44/0.52/97.64	0.88/0.51/94.80	0.48/0.53/96.43
MMD; Li H. et al. (2018)	0.12/0.55/97.53	0.43/0.51/96.34	0.92/0.51/96.59	0.49/0.53/96.82
DANN; Ganin et al. (2016)	**0.92**/0.97/53.73	0.01/0.76/69.87	0.40/0.82/51.65	0.44/0.85/58.42
CDANN; Li Y. et al. (2018)	0.00/0.95/50.81	0.00/0.75/69.98	0.30/0.77/58.47	0.10/0.83/59.76
DDG; Zhang et al. (2022)	0.18/0.52/96.19	0.40/0.51/90.48	0.78/0.51/89.96	0.45/**0.51**/92.21
MBDG; Robey et al. (2021)	0.16/**0.51**/97.39	0.44/**0.50**/97.97	0.95/**0.50**/96.41	**0.52/0.51**/97.26
DAD; Peng et al. (2024)	0.12/0.53/96.75	0.44/0.52/96.31	0.92/0.54/96.95	0.49/0.53/96.67
TD-BLS; Zhang et al. (2025)	0.11/0.53/96.94	0.42/**0.50**/95.50	0.93/0.54/96.79	0.48/0.52/96.41
DLRE; Zhao et al. (2025)	0.13/0.52/95.51	0.45/0.51/94.85	0.93/0.54/95.64	0.51/0.53/95.34
DDG-FC; Zhang et al. (2022)	0.14/**0.51**/94.69	0.45/0.51/89.73	0.81/0.51/88.19	0.47/**0.51**/90.87
MBDG-FC; Robey et al. (2021)	0.13/**0.51**/82.85	0.45/0.51/89.83	**0.98/0.50**/84.96	**0.52/0.51**/85.88
FFDGUA	0.13/0.53/**97.93**	**0.46**/0.52/**98.11**	**0.98**/0.51/**96.82**	**0.52**/0.52/**97.62**

Bold is the best; underline is the second best.

**Table 13:** Unsupervised domain adaptation performance on FairFace.

Methods	*DP* ↑/*AUC*_*fair*_ ↓/*Acc* ↑			
	(B, 0.91)	(E, 0.87)	(I, 0.58)	(W, 0.49)
CORAL; Sun and Saenko (2016)	0.78/0.51/92.78	0.42/0.52/92.87	0.58/0.51/90.24	0.29/0.52/89.43
MMD; Li H. et al. (2018)	0.73/0.51/90.76	0.35/0.52/92.92	0.59/0.51/87.51	0.34/0.52/86.91
DANN; Ganin et al. (2016)	0.69/0.86/84.15	**0.93**/0.75/52.88	**0.93**/0.77/68.12	**0.92**/0.76/54.72
CDANN; Li Y. et al. (2018)	0.51/0.79/80.94	0.56/0.90/79.31	0.39/0.92/85.63	0.84/0.76/58.25
DDG; Zhang et al. (2022)	0.69/0.52/90.95	0.34/**0.51**/94.69	0.46/0.51/90.60	0.29/**0.50**/90.78
MBDG; Robey et al. (2021)	0.83/0.52/92.27	0.31/**0.51**/94.41	0.48/0.51/91.90	0.44/0.51/91.22
DAD; Peng et al. (2024)	0.91/0.52/**93.40**	0.21/0.54/94.40	0.56/0.52/91.43	0.37/0.53/92.32
TD-BLS; Zhang et al. (2025)	0.90/0.54/92.71	0.32/0.58/94.22	0.51/0.54/90.89	0.33/0.55/91.63
DLRE; Zhao et al. (2025)	**0.99**/0.54/93.22	0.35/0.58/95.05	0.51/0.53/91.54	0.34/0.54/**92.36**
DDG-FC; Zhang et al. (2022)	0.42/**0.50**/90.75	0.43/**0.51**/92.54	0.61/0.51/85.92	0.40/0.51/85.89
MBDG-FC; Robey et al. (2021)	0.58/0.52/92.62	0.58/**0.51**/93.48	0.84/**0.50**/89.79	0.43/0.51/90.46
FFDGUA	0.74/**0.50**/92.69	0.60/**0.51/95.48**	0.65/**0.50/92.14**	0.54/0.51/91.20
**Methods**	*DP* ↑/*AUC*_*fair*_ ↓/*Acc* ↑			
	**(L, 0.48)**	**(M, 0.87)**	**(S, 0.39)**	**Avg**
CORAL; Sun and Saenko (2016)	0.49/0.52/92.38	0.33/0.52/90.68	0.64/0.52/92.65	0.51/0.52/91.57
MMD; Li H. et al. (2018)	0.47/0.52/87.91	0.43/0.51/85.21	0.63/**0.51**/89.88	0.51/**0.51**/88.73
DANN; Ganin et al. (2016)	0.85/0.93/85.23	0.70/0.75/70.51	0.54/0.86/88.62	**0.80**/0.81/72.03
CDANN; Li Y. et al. (2018)	**0.94**/0.82/57.29	**0.84**/0.74/69.82	**0.90**/0.75/64.13	0.71/0.81/70.77
DDG; Zhang et al. (2022)	0.38/0.52/91.19	0.28/0.51/91.40	0.48/0.52/89.98	0.42/**0.51**/91.37
MBDG; Robey et al. (2021)	0.65/**0.51**/92.65	0.34/0.52/91.21	0.64/**0.51**/92.51	0.53/**0.51**/92.31
DAD; Peng et al. (2024)	0.12/0.56/90.94	0.66/0.53/92.33	0.22/0.53/91.99	0.44/0.53/92.40
TD-BLS; Zhang et al. (2025)	0.14/0.58/90.50	0.69/**0.50**/92.12	0.23/0.53/91.43	0.45/0.55/91.92
DLRE; Zhao et al. (2025)	0.22/0.55/91.37	0.63/0.51/**92.58**	0.25/0.55/92.26	0.47/0.54/92.63
DDG-FC; Zhang et al. (2022)	0.53/**0.51**/88.70	0.38/0.51/90.98	0.66/**0.51**/90.04	0.49/**0.51**/89.26
MBDG-FC; Robey et al. (2021)	0.59/**0.51**/90.03	0.55/0.52/86.97	0.72/**0.51**/91.76	0.61/**0.51**/90.73
FFDGUA	0.80/**0.51/92.78**	0.56/0.51/92.34	0.79/**0.51/93.61**	0.67/**0.51/92.89**

Bold is the best; underline is the second best.

#### 5.3.5 Ablation studies

**Figure 13:**
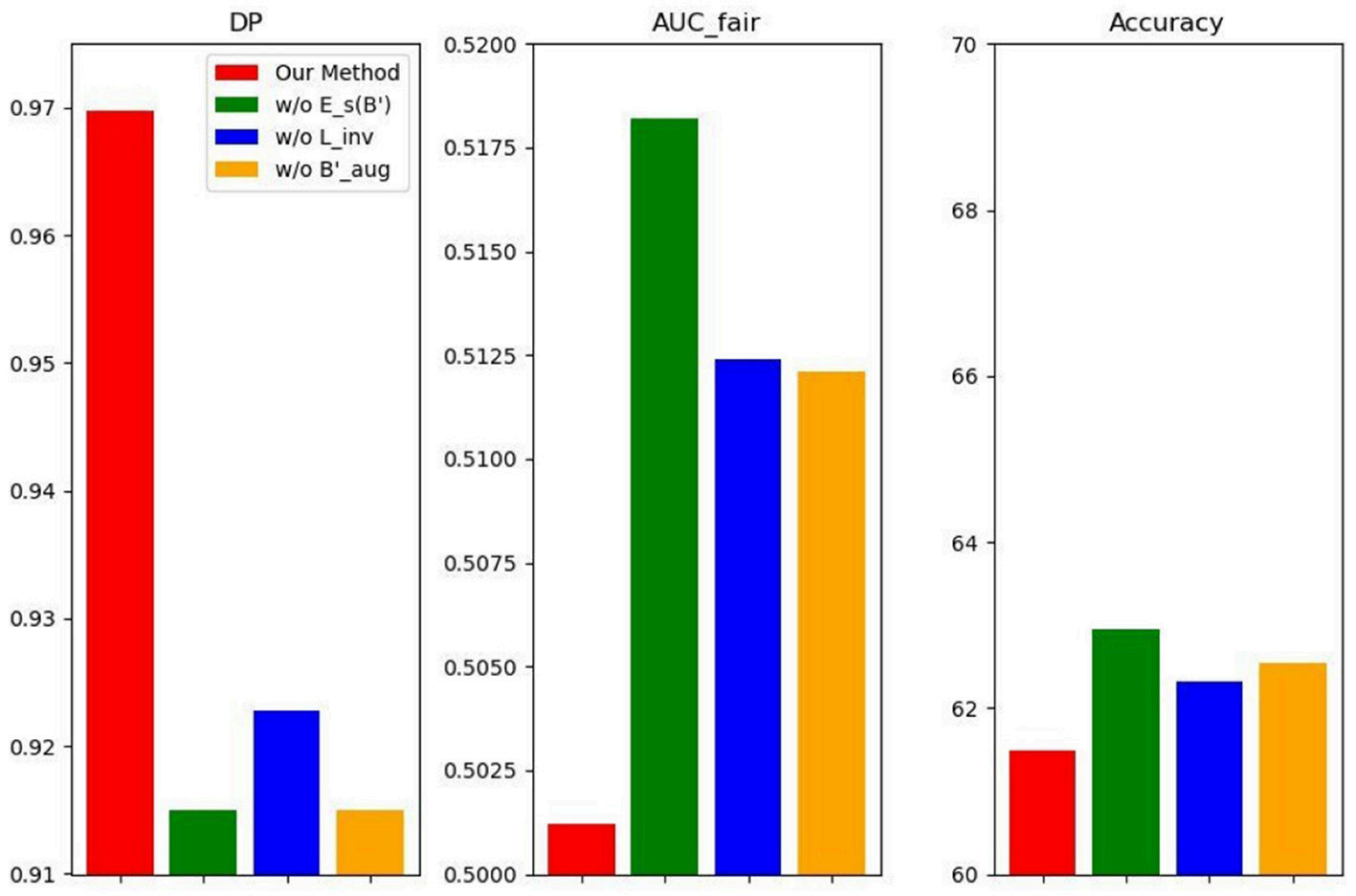
Unsupervised domain adaptation ablation study on NYSF, with averaged results plotted across all domains.

We conduct three ablation studies to evaluate the robustness of FFDGUA on the NYSFdataset. In FFDGUA w/o $Es\left({ℬ}^{\prime }\right)$, we adopt a similar approach to domain generalization while also incorporating unlabeled samples from the target domain to generate additional unlabeled samples. In FFDGUA w/o ℒinv, we omit the generation of ${ℬ}_{aug}^{\prime }$in Algorithm 2 and do not compute ${ℒ}_{inv}$. In FFDGUA w/o ${ℬ}^{\prime }aug$, we also exclude the generation of ${ℬ}_{aug}^{\prime }$in Algorithm 2, but we calculate ${ℒ}_{inv}$ using ℬ and ${ℬ}_{aug}$. The performance of these ablation studies is presented in Figure 13. As shown in Tables 14–17, the results indicate that disentangling data into three distinct latent factors, content, style, and sensitive factors, alongside a model structure explicitly designed to account for these factors, improves generalization performance under covariate and dependence shifts. Moreover, generating data in the target domains with randomized fairness dependence patterns emerges as an effective strategy for ensuring fairness invariance.

**Table 14:** Unsupervised domain adaptation ablation studies results on ccMNIST.

Methods	*DP* ↑/*AUC*_*fair*_ ↓/*Acc* ↑			
	(R, 0.11)	(G, 0.43)	(B, 0.87)	Avg
FFDGUA w/o $Es\left({ℬ}^{\prime }\right)$	0.12/0.53/98.32	0.46/0.50/98.11	0.96/0.52/98.06	0.51/0.52/98.16
FFDGUA w/o ℒinv	0.12/0.53/98.04	0.46/0.52/97.02	0.99/0.50/98.17	0.52/0.52/97.74
FFDGUA w/o ${ℬ}^{\prime }aug$	0.14/0.54/97.97	0.45/0.51/98.04	0.98/0.51/97.14	0.52/0.52/97.71

**Table 15:** Unsupervised domain adaptation ablation studies results on FairFace.

Methods	*DP* ↑/*AUC*_*fair*_ ↓/*Acc* ↑			
	(B, 0.91)	(E, 0.87)	(I, 0.58)	(W, 0.49)
FFDGUA w/o $Es\left({ℬ}^{\prime }\right)$	0.81/0.51/93.15	0.48/0.51/95.48	0.41/0.52/91.56	0.31/0.52/91.90
FFDGUA w/o ℒinv	0.97/0.51/92.78	0.62/0.51/95.58	0.93/0.50/91.21	0.46/0.51/92.33
FFDGUA w/o ${ℬ}^{\prime }aug$	0.99/0.51/93.10	0.58/0.51/95.59	0.71/0.50/91.66	0.45/0.52/91.84
**Methods**	*DP* ↑/*AUC*_*fair*_ ↓/*Acc* ↑			
	**(L, 0.48)**	**(M, 0.87)**	**(S, 0.39)**	**Avg**
FFDGUA w/o $Es\left({ℬ}^{\prime }\right)$	0.51/0.52/93.01	0.39/0.50/92.52	0.72/0.51/93.42	0.52/0.51/93.00
FFDGUA w/o ℒinv	0.76/0.51/92.11	0.66/0.52/91.40	0.77/0.51/94.02	0.74/0.51/92.78
FFDGUA w/o ${ℬ}^{\prime }aug$	0.67/0.51/92.21	0.56/0.51/92.17	0.82/0.51/93.61	0.68/0.51/92.88

**Table 16:** Unsupervised domain adaptation ablation studies results on YFCC100M-FDG.

Methods	*DP* ↑/*AUC*_*fair*_ ↓/*Acc* ↑			
	(*d*_0_, 0.73)	(*d*_1_, 0.84)	(*d*_2_, 0.72)	Avg
FFDGUA w/o $Es\left({ℬ}^{\prime }\right)$	0.85/0.50/99.70	0.93/0.50/93.42	0.95/0.51/90.94	0.91/0.50/94.69
FFDGUA w/o ℒinv	0.85/0.54/84.59	0.93/0.53/95.32	0.93/0.52/95.96	0.91/0.53/91.96
FFDGUA w/o ${ℬ}^{\prime }aug$	0.96/0.50/81.70	0.98/0.50/82.73	0.95/0.51/88.52	0.96/0.50/84.32

**Table 17:** Unsupervised domain adaptation ablation studies results on NYSF.

Methods	*DP* ↑/*AUC*_*fair*_ ↓/*Acc* ↑		
	(R, 0.93)	(B, 0.85)	(M, 0.81)
FFDGUA w/o $Es\left({ℬ}^{\prime }\right)$	0.99/0.50/65.93	0.93/0.51/57.61	0.91/0.51/62.05
FFDGUA w/o ℒinv	0.94/0.51/63.14	0.95/0.50/58.84	0.94/0.50/61.86
FFDGUA w/o ${ℬ}^{\prime }aug$	0.95/0.51/64.38	0.92/0.50/59.14	0.93/0.50/61.15
**Methods**	*DP* ↑/*AUC*_*fair*_ ↓/*Acc* ↑		
	**(Q, 0.59)**	**(S, 0.62)**	**Avg**
FFDGUA w/o $Es\left({ℬ}^{\prime }\right)$	0.95/0.50/65.39	0.81/0.56/63.73	0.92/0.52/62.94
FFDGUA w/o ℒinv	0.85/0.52/63.96	0.93/0.54/63.83	0.92/0.51/62.32
FFDGUA w/o ${ℬ}^{\prime }aug$	0.84/0.52/64.74	0.92/0.53/63.29	0.91/0.51/62.54

### 5.4 Comparing experiment results between two setting

The experimental results demonstrate that the unified framework FFDGUA achieves consistently strong performance across both domain generalization (DG) and unsupervised domain adaptation (UDA) tasks. For the ccMNISTdataset, both DG and UDA yield competitive accuracy (96.76% and 97.62%, respectively), and fairness performance on *AUC*_*fair*_ (0.66 vs. 0.52) and *DP* (0.71 vs. 0.52). In FairFace, UDA and DG still have similar fairness performance, where UDA achieves a lower demographic parity (0.67 vs. 0.7) and lower *AUC*_*fair*_ (0.51 vs. 0.58), while maintaining comparable accuracy (92.89% vs. 98.42%). On the YFCC100M-FDGdataset, UDA delivers competitive fairness (*DP* of 0.91 vs. 0.92; *AUC*_*fair*_ of 0.51 vs. 0.53) and a significant improvement in accuracy (89.51% vs. 83.12%). For the tabular NYSF dataset, although the accuracy gains from UDA are modest (61.49% vs. 59.95%), it maintains equivalent *DP* (0.97 vs. 0.97) and slightly better *AUC*_*fair*_ of (0.5 vs. 0.51). Overall, compared to DG, the UDA setting demonstrates consistent fairness improvements while achieving comparable or better accuracy across all benchmark datasets. UDA's performance advantage originates from its effective utilization of unlabeled target domain data, which enables more comprehensive learning of domain-specific variations while preserving fairness constraints. The integration of target domain style factors into the training process enhances the robustness of the classifier to unseen shifts, enabling more accurate and equitable predictions across domains.

## 6 Conclusion

This study extends the work in Zhao et al. (2024) by proposing a unified framework for fairness-aware learning across both Domain Generalization (DG) and Unsupervised Domain Adaptation (UDA). While Zhao et al. (2024) focused solely on DG, our framework expands its scope to UDA by effectively leveraging both labeled source domains and unlabeled target domains. This dual capability enables robust and fair learning across diverse and challenging scenarios. The comprehensive experimental results validate the proposed framework's effectiveness. Across four datasets, our framework consistently outperforms state-of-the-art methods in terms of accuracy and fairness metrics, demonstrating its superior ability to mitigate biases associated with sensitive attributes under domain shifts. Despite its strengths, the study is not without limitations. The current implementation requires simultaneous access to multiple labeled source domains with sensitive attribute annotations, which is a strong assumption that may not hold in real-world scenarios where data collection is decentralized. Additionally, the computational complexity of disentanglement and fairness-aware optimization could be a bottleneck in resource-constrained environments. Future research can build upon this framework by exploring efficient algorithms to reduce computational overhead and investigating methods to relax the assumptions on labeled data and sensitive attribute availability. Furthermore, extending the framework to handle multi-label classification and continuous sensitive attributes could enhance its applicability to broader settings.

## Data Availability

The original contributions presented in the study are included in the article/supplementary material, further inquiries can be directed to the corresponding author.

## Footnotes

1 The ρ operator follows the same definition as in Equation 1, replacing Ŷ with *Y*.

## References

[B1] An B. Che Z. Ding M. Huang F. (2022). Transferring fairness under distribution shifts via fair consistency regularization. Adv. Neural Inf. Process. Syst. 35, 32582–32597. doi: 10.52202/068431-2361

[B2] Arjovsky M. Bottou L. Gulrajani I. Lopez-Paz D. (2019). Invariant risk minimization. arXiv [preprint] arXiv:1907.02893. doi: 10.48550/arXiv.1907.02893

[B3] Blanchard G. Lee G. Scott C. (2011). “Generalizing from several related classification tasks to a new unlabeled sample,” in Advances in Neural Information Processing Systems (Red Hook, NY: Curran Associates, Inc.), 24.

[B4] Calders T. Karim A. Kamiran F. Ali W. Zhang X. (2013). “Controlling attribute effect in linear regression,” in ICDM (Dallas, TX: IEEE).

[B5] Creager E. Jacobsen J.-H. Zemel R. (2021). “Environment inference for invariant learning,” in International Conference on Machine Learning (New York, NY: PMLR), 2189–2200.

[B6] Dwork C. Hardt M. Pitassi T. Reingold O. Zemel R. (2011). Fairness Through Awareness. New York, NY: ACM.

[B7] Ganin Y. Ustinova E. Ajakan H. Germain P. Larochelle H. Laviolette F. . (2016). Domain-adversarial training of neural networks. J. Mach. Learn. Res. 17, 2096–2030.

[B8] Giguere S. Metevier B. Brun Y. da Silva B. C. Thomas P. S. Niekum S. (2022). “Fairness guarantees under demographic shift,” in Proceedings of the 10th International Conference on Learning Representations (ICLR) (OpenReview).

[B9] Goodfellow I. Pouget-Abadie J. Mirza M. Xu B. Warde-Farley D. Ozair, S. . (2020). Generative adversarial networks. Commun. ACM 63, 139–144. doi: 10.1145/3422622

[B10] Gulrajani I. Lopez-Paz D. (2020). In search of lost domain generalization. arXiv [preprint] arXiv:2007.01434. doi: 10.48550/arXiv.2007.01434

[B11] He K. Zhang X. Ren S. Sun J. (2016). “Deep residual learning for image recognition,” in In Proceedings of the IEEE Conference on Computer Vision and Pattern Recognition (Las Vegas, NV: IEEE), 770–778.

[B12] Hoffman J. Tzeng E. Park T. Zhu J.-Y. Isola P. Saenko K. . (2018). “Cycada: Cycle-consistent adversarial domain adaptation,” in International Conference on Machine Learning (New York, NY: PMLR), 1989–1998.

[B13] Huang X. Liu M.-Y. Belongie S. Kautz J. (2018). “Multimodal unsupervised image-to-image translation,” in Proceedings of the European Conference on Computer Vision (ECCV) (Munich: Springer), 172–189.

[B14] Karkkainen K. Joo J. (2021). “FairFace: Face attribute dataset for balanced race, gender, and age for bias measurement and mitigation,” in Proceedings of the IEEE/CVF Winter Conference on Applications of Computer Vision (WACV) (Waikoloa, HI: IEEE), 1548–1558.

[B15] Koh P. W. Sagawa S. Marklund H. Xie S. M. Zhang M. Balsubramani A. . (2021). “Wilds: a benchmark of in-the-wild distribution shifts,” in International Conference on Machine Learning (New York, NY: PMLR), 5637–5664.

[B16] LeCun Y. Bottou L. Bengio Y. Haffner P. (1998). Gradient-based learning applied to document recognition. Proc. IEEE 86, 2278–2324. doi: 10.1109/5.726791

[B17] Li D. Yang Y. Song Y.-Z. Hospedales T. (2018). “Learning to generalize: meta-learning for domain generalization,” in Proceedings of the AAAI Conference on Artificial Intelligence (New Orleans, LA: AAAI Press), 3490–3497.

[B18] Li H. Pan S. J. Wang S. Kot A. C. (2018). “Domain generalization with adversarial feature learning,” in Proceedings of the IEEE Conference on Computer Vision and Pattern Recognition (Salt Lake City, UT: IEEE), 5400–5409.

[B19] Li Y. Tian X. Gong M. Liu Y. Liu T. Zhang K. . (2018). “Deep domain generalization via conditional invariant adversarial networks,” in Proceedings of the European Conference on Computer Vision (ECCV) (Munich: Springer), 624–639.

[B20] Li Y. Yang Y. Zhou W. Hospedales T. (2019). “Feature-critic networks for heterogeneous domain generalization,” in International Conference on Machine Learning (New York, NY: PMLR), 3915–3924.

[B21] Ling C. X. Huang J. Zhang H. (2003). AUC: a statistically consistent and more discriminating measure than accuracy. IJCAI 3, 519–524.

[B22] Liu M.-Y. Breuel T. Kautz J. (2017). “Unsupervised image-to-image translation networks,” in Advances in Neural Information Processing Systems (Red Hook, NY: Curran Associates, Inc.), 30.

[B23] Lohaus M. Perrot M. Von Luxburg U. (2020). “Too relaxed to be fair,” in International Conference on Machine Learning (New York, NY: PMLR), 6360–6369.

[B24] Nguyen A. Schwab D. J. Ngampruetikorn V. (2024). Generalization vs. specialization under concept shift. arXiv [preprint] arXiv:2409.15582. doi: 10.48550/arXiv.2409.15582

[B25] Oh C. Won H. So J. Kim T. Kim Y. Choi H. . (2022). “Learning fair representation via distributional contrastive disentanglement,” in Proceedings of the 28th ACM SIGKDD Conference on Knowledge Discovery and Data Mining (Washington, DC: ACM), 1295–1305.

[B26] Peng D. Ke Q. Ambikapathi A. Yazici Y. Lei Y. Liu J. (2024). Unsupervised domain adaptation via domain-adaptive diffusion. IEEE Trans. Image Proc. 33, 4245–4260. doi: 10.1109/TIP.2024.3424985 39008383

[B27] Peng X. Bai C. Xia X. Huang Z. Saenko K. Wang B. (2019). “Moment matching for multi-source domain adaptation,” in International Conference on Computer Vision (Seoul: IEEE), 1406–1415. doi: 10.1109/ICCV.2019.00149

[B28] Pham T.-H. Zhang X. Zhang P. (2023). “Fairness and accuracy under domain generalization,” in Proceedings of the International Conference on Learning Representations (Kigali: ICLR). 37292471

[B29] Quadrianto N. Sharmanska V. Thomas O. (2019). “Discovering fair representations in the data domain,” in Proceedings of the IEEE/CVF Conference on Computer Vision and Pattern Recognition (Long Beach, CA: IEEE), 8227–8236.

[B30] Robey A. Pappas G. J. Hassani H. (2021). “Model-based domain generalization,” in Advances in Neural Information Processing Systems (Red Hook, NY: Curran Associates, Inc.), 34, 20210–20229.

[B31] Roh Y. Lee K. Whang S. E. Suh C. (2023). “Improving fair training under correlation shifts,” in ICML (Honolulu, HI: PMLR).

[B32] Rostami M. Galstyan A. (2023). “Overcoming concept shift in domain-aware settings through consolidated internal distributions,” in Proceedings of the AAAI Conference on Artificial Intelligence (Washington, DC: AAAI Press), 37, 9623–9631.

[B33] Sagawa S. Koh P. W. Hashimoto T. B. Liang P. (2020). “Distributionally robust neural networks,” in International Conference on Learning Representations (OpenReview).

[B34] Shao M. Li D. Zhao C. Wu X. Lin Y. Tian Q. (2024). Supervised algorithmic fairness in distribution shifts: a survey. arXiv [preprint] arXiv:2402.01327. doi: 10.48550/arXiv.2402.01327

[B35] Sun B. Saenko K. (2016). “Deep coral: correlation alignment for deep domain adaptation,” in European Conference on Computer Vision (Amsterdam: Springer), 443–450.

[B36] Tachet des Combes R. Zhao H. Wang Y.-X. Gordon G. J. (2020). “Domain adaptation with conditional distribution matching and generalized label shift,” in Advances in Neural Information Processing Systems (Red Hook, NY: Curran Associates, Inc.), 33, 19276–19289.

[B37] Thomee B. Shamma D. A. Friedland G. Elizalde B. Ni K. Poland D. . (2016). YFCC100M: The new data in multimedia research. Commun. ACM 59, 64–73. doi: 10.1145/2812802

[B38] Vapnik V. (1999). The Nature of Statistical Learning Theory. Berlin: Springer Science & Business Media.

[B39] Volpi R. Larlus D. Rogez G. (2021). “Continual adaptation of visual representations via domain randomization and meta-learning,” in Proceedings of the IEEE/CVF Conference on Computer Vision and Pattern Recognition (Nashville, TN: IEEE), 4443–4453.

[B40] Volpi R. Namkoong H. Sener O. Duchi J. C. Murino V. Savarese S. (2018). “Generalizing to unseen domains via adversarial data augmentation,” in Advances in Neural Information Processing Systems (Red Hook, NY: Curran Associates, Inc.), 31.

[B41] Wang Z. Qinami K. Karakozis I. C. Genova K. Nair P. Hata K. . (2020). “Towards fairness in visual recognition: Effective strategies for bias mitigation,” in Proceedings of the IEEE/CVF Conference on Computer Vision and Pattern Recognition (Seattle, WA: IEEE), 8919–8928.

[B42] Wu Y. Zhang L. Wu X. (2019). “On convexity and bounds of fairness-aware classification,” in The World Wide Web Conference (San Francisco, CA: ACM), 3356–3362.

[B43] Xiao Z. Wang H. Ye Y. Ye W. Chen H. Chen G. . (2026). LADA: Label disambiguation and domain-aware learning for domain generalization. Mach. Learn. 115, 53. doi: 10.1007/s10994-025-06977-w

[B44] Yan S. Song, H Li N. Zou L. Ren L. (2020). Improve unsupervised domain adaptation with mixup training. arXiv [preprint] arXiv:2001.00677. doi: 10.48550/arXiv.2001.00677

[B45] Zhang H. Zhang Y.-F. Liu W. Weller A. Schölkopf B. Xing E. P. (2022). “Towards principled disentanglement for domain generalization,” in Proceedings of the IEEE/CVF Conference on Computer Vision and Pattern Recognition (New Orleans, LA: IEEE), 8024–8034. doi: 10.48550/arXiv.2111.13839

[B46] Zhang L. Yu Z. Yang K. Wang B. Chen C. P. (2025). Transferable and discriminative broad network for unsupervised domain adaptation. Knowl.-Based Syst. 315:113297. doi: 10.1016/j.knosys.2025.113297

[B47] Zhao C. Chen F. (2019). “Rank-based multi-task learning for fair regression,” in IEEE International Conference on Data Mining (ICDM) (Beijing: IEEE).

[B48] Zhao C. Jiang K. Wu X. Wang H. Khan L. Grant C. . (2024). “Algorithmic fairness generalization under covariate and dependence shifts simultaneously,” in Proceedings of the 30th ACM SIGKDD Conference on Knowledge Discovery and Data Mining (Barcelona: ACM), 4419–4430.

[B49] Zhao C. Mi F. Wu X. Jiang K. Khan L. Grant C. . (2023). “Towards fair disentangled online learning for changing environments,” in Proceedings of the 29th ACM SIGKDD Conference on Knowledge Discovery and Data Mining (Long Beach, CA: ACM), 3480–3491.

[B50] Zhao D. Yang F. Hu T. Wei X. Zhao C. Lu Y. (2025). Dual-level redundancy elimination for unsupervised domain adaptation. Expert Syst. Appl. 276:127090. doi: 10.1016/j.eswa.2025.127090

[B51] Zhao H. Zhang S. Wu G.-J. Moura J. M. Costeira J. P. Gordon G. J. (2018). “Adversarial multiple source domain adaptation,” in Advances in Neural Information Processing Systems (Red Hook, NY: Curran Associates, Inc.), 855–865.

[B52] Zhou K. Yang Y. Hospedales T. Xiang T. (2020). “Learning to generate novel domains for domain generalization,” in Computer Vision-ECCV 2020: 16th European Conference (Glasgow: Springer), 561–578.

[B53] Zhou K. Yang Y. Qiao Y. Xiang T. (2021). Domain generalization with mixstyle. arXiv [preprint] arXiv:2104.02008. doi: 10.48550/arXiv.2104.02008

